# Supplementary material for: Structural conservation of HBV-like capsid proteins over hundreds of millions of years despite the shift from non-enveloped to enveloped life-style
Source: Nat Commun. 2023 Mar 22;14:1574. doi: 10.1038/s41467-023-37068-w (PMC10033635; doi:10.1038/s41467-023-37068-w)
Supplement: Supplementary file 1 — Supplementary Information [file 41467_2023_37068_MOESM1_ESM.pdf]

Supporting Information for:

Structural conservation of HBV-like capsid proteins over hundreds of millions of years despite the shift from non-enveloped to enveloped life-style

Sara Pfister[1], Julius Rabl[2], Thomas Wiegand[1,a,b], Simone Mattei[3], Alexander A. Malär[1], Lauriane Lecoq[4], Stefan Seitz[5,6], Ralf Bartenschlager[6], Anja Böckmann*[4], Michael Nassal*[7], Daniel Boehringer*[2], and Beat H. Meier*[1]

[1] Physical Chemistry, ETH Zurich, 8093 Zurich, Switzerland

[2] Cryo-EM Knowledge hub, ETH Zurich, 8093 Zurich, Switzerland

[3] EMBL Imaging Centre, European Molecular Biology Laboratory, EMBL Heidelberg, 69117 Heidelberg, Germany

[4] Molecular Microbiology and Structural Biochemistry, UMR 5086 CNRS, Université de Lyon, 69367 Lyon, France

[5] Division of Virus-Associated Carcinogenesis (F170), German Cancer Research Center (DKFZ), 69120 Heidelberg, Germany

[6] Department of Infectious Diseases, Molecular Virology, University of Heidelberg, 69120 Heidelberg, Germany

[7] Department of Medicine II / Molecular Biology, University of Freiburg, Freiburg im Breisgau, Germany

[a] current address: Max-Planck-Institute for Chemical Energy Conversion, Stiftstr. 34-36, 45470 Mülheim an der Ruhr, Germany

[b] current address: Institute of Technical and Macromolecular Chemistry, RWTH Aachen University, Worringerweg 2, 52074 Aachen, Germany

Table S 1: Solid-state NMR spectra for ACNDV capsid assignment at pH 7.5

Carbon-detected experiments [a), b)]	Proton-detected experiments [a)]
2D DARR 20 ms	2D hNH
2D NCA	2D hCH
2D NCO	3D hCANH
3D NCACB	3D hNCAH
3D NCACX	3D hCAcoNH
3D NCOCX	3D hNcoCAH
3D CANCO	
3D CCC	
3D CANcoCA	
3D NcoCACB	

[a)] Experimental parameters are listed in Table S 3. [b)] The carbon-detected experiments listed here were used for calculation of secondary chemical shifts.

Table S 2: Solid-state NMR assignment completeness ACNDV capsid at pH 7.5

Category	Assignment completeness for all 175 residues (%) [a)]	Assignment completeness for residues 3-134 (%) [b)]
Backbone	57	76
Backbone + H + HA	52	70
Sidechain non-H	42	55
N	58	77
C	57	75
CA	58	77
CB	60	75
H	48	62
HA	42	59

[a)] Our ACNDV capsid protein construct is made up of 175 residues. A glycine was inserted as second residue for higher expression yields. Only unambiguous assignments are considered. [b)] The first residue and the C-terminus are flexible and were not modeled in the cryo-EM structure. Only unambiguous assignments are considered.

Table S 3: Parameters of NMR experiments

	**DARR pH 7.5**	**DARR pH 7.5 denaturation**	**DARR pH 5.5**	**DARR pH 9**
MAS frequency / kHz	17	17	17	17
Field / T	20	20	20	20
Number of scans	12	8	20	8
t1 increment	2000	2560	2560	3072
Sweep width (t1) / ppm	467.7	467.7	467.7	467.7
Acquisition time (t1) / ms	10.0	12.8	12.8	15.4
t2 increments	3072	3072	3072	3072
Sweep width (t2) / ppm	467.7	467.7	467.7	467.7
Acquisition time (t2) / ms	15.4	15.4	15.4	15.4
^{1}H decoupling / kHz	SPINAL64 / 90	SPINAL64 / 90	SPINAL64 / 90	SPINAL64 / 90
Interscan delay / s	2.7	2.7	2.7	2.7
Experiment time	18.5 h	16 h	1 d 16 h	19 h
**Transfer 1**	**HC-CP**	**HC-CP**	**HC-CP**	**HC-CP**
Field / kHz	60 (^{1}H) / 41 (^{13}C)	60 (^{1}H) / 43 (^{13}C)	60 (^{1}H) / 41 (^{13}C)	60 (^{1}H) / 42 (^{13}C)
Shape	Tangent ^{1}H	Tangent ^{1}H	Tangent ^{1}H	Tangent ^{1}H
^{13}C carrier / ppm	100	100	100	100
Time / ms	0.6	0.6	0.5	0.55
**Transfer 2**	**DARR**	**DARR**	**DARR**	**DARR**
Field / kHz	17 (^{1}H)	17 (^{1}H)	17 (^{1}H)	17 (^{1}H)
^{13}C carrier / ppm	100	100	100	100
Time / ms	20	20	20	20
Window function	QSINE SSB 2.5	QSINE SSB 2.5	QSINE SSB 2.5	QSINE SSB 2.5
Sample prep [a)]	a.	f.	c.	d.

[a)] Expression protocols in SI section listed for a. – k.

	DARR lysine	DARR SEC
MAS frequency / kHz	17	17
Field / T	20	20
Number of scans	32	12
t1 increment	2560	2560
Sweep width (t1) / ppm	467.7	467.7
Acquisition time (t1) / ms	12.8	12.8
t2 increments	3072	3072
Sweep width (t2) / ppm	467.7	467.7
Acquisition time (t2) / ms	15.4	15.4
^{1}H decoupling / kHz	SPINAL64 / 90	SPINAL64 / 90
Interscan delay / s	2.7	2.7
Experiment time	2 d 20 h	20 h
**Transfer 1**	**HC-CP**	**HC-CP**
Field / kHz	60 (^{1}H) / 43 (^{13}C)	60 (^{1}H) / 44 (^{13}C)
Shape	Tangent ^{1}H	Tangent ^{1}H
^{13}C carrier / ppm	100	100
Time / ms	0.5	0.5
**Transfer 2**	**DARR**	**DARR**
Field / kHz	17 (^{1}H)	17 (^{1}H)
^{13}C carrier / ppm	100	100
Time / ms	20	20
Window function	QSINE SSB 2.5	QSINE SSB 2.5
TDeff	-	TD2eff 1862
Sample prep [a)]	e.	h.

[a)] Expression protocols in SI section listed for a. – k.

	NCACB	NCACX	CANCO	NCOCX
MAS frequency / kHz	17	17	17	17
Field / T	20	20	20	20
Number of scans	8	16	16	32
t1 increment	82	68	102	60
Sweep width (t1) / ppm	55.0	55.0	35.0	55.0
Acquisition time (t1) / ms	8.7	7.2	6.8	6.3
t2 increments	118	120	60	104
Sweep width (t2) / ppm	35.0	35.0	55.0	30.0
Acquisition time (t2) / ms	7.9	8.0	6.3	8.1
t3 increments	2304	2304	2304	2304
Sweep width (t3) / ppm	467.7	467.7	467.7	467.7
Acquisition time (t3) / ms	11.5	11.5	11.5	11.5
^{1}H decoupling / kHz	SPINAL64 / 90	SPINAL64 / 90	SPINAL64 / 90	SPINAL64 / 90
Interscan delay / s	3.0	3.0	3.0	3.0
Experiment time	2 d 17 h	4 d 16 h	3 d 10 h	7 d 4 h
**Transfer 1**	**HN-CP**	**HN-CP**	**HCA-CP**	**HN-CP**
Field / kHz	60 (^{1}H) / 45 (^{15}N)	60 (^{1}H) / 45 (^{15}N)	60 (^{1}H) / 41 (^{13}C)	60 (^{1}H) / 45 (^{15}N)
Shape	Tangent ^{1}H	Tangent ^{1}H	Tangent ^{1}H	Tangent ^{1}H
Carrier / ppm	120 (^{15}N)	120 (^{15}N)	61.2 (^{13}C)	120 (^{15}N)
Time / ms	0.8	0.8	0.6	0.6
**Transfer 2**	**NCA-CP**	**NCA-CP**	**CAN-CP**	**NCO-CP**
Field / kHz	10.7 (^{15}N) / 6.0 (^{13}C)	10.7 (^{15}N) / 6.0 (^{13}C)	6 (^{13}C) / 10.5 (^{15}N)	20.8 (^{15}N) / 6.0 (^{13}C)
Shape	Tangent ^{13}C	Tangent ^{13}C	Tangent ^{13}C	Tangent ^{13}C
Carrier / ppm	61.2 (^{13}C)	61.2 (^{13}C)	61.2 (^{13}C)	176.7 (^{13}C)
Time / ms	6.4	6.4	6.5	5.0
**Transfer 3**	**DREAM**	**DARR**	**NCO-CP**	**DARR**
Field / kHz	7.3 (^{13}C)	17 (^{1}H)	20.8 (^{15}N) / 6.0 (^{13}C)	17 (^{1}H)
Shape	Tangent ^{13}C	-	Tangent ^{13}C	-
Carrier / ppm	57.0 (^{13}C)	178.5 (^{13}C)	176.7 (^{13}C)	176.7 (^{13}C)
Time / ms	1.75	70.0	5.0	70.0
Window function	QSINE SSB 2.5	QSINE SSB 2.5	QSINE SSB 2.5	QSINE SSB 2.5
Sample prep [a)]	a.	a.	a.	a.

[a)] Expression protocols in SI section listed for a. – k.

	NCO	NCA	CANcoCA	NcoCACB
MAS frequency / kHz	17	17	17	17
Field / T	20	20	20	20
Number of scans	16	16	16	16
t1 increment	1536	1536	112	70
Sweep width (t1) / ppm	773.7	773.7	35.0	55.0
Acquisition time (t1) / ms	11.5	11.5	7.5	7.4
t2 increments	3072	3072	60	114
Sweep width (t2) / ppm	467.7	467.7	55.0	35.0
Acquisition time (t2) / ms	15.4	15.4	6.3	7.6
t3 increments	-	-	2304	3072
Sweep width (t3) / ppm	-	-	467.	467.7
Acquisition time (t3) / ms	-	-	11.5	15.4
^{1}H decoupling / kHz	SPINAL64 / 90	SPINAL64 / 90	SPINAL64 / 90	SPINAL64 / 90
Interscan delay / s	2.7	3.0	3.2	3.2
Experiment time	19 h	23 h	4 d 1 h	4 d 19 h
**Transfer 1**	**HN-CP**	**HN-CP**	**HCA-CP**	**HN-CP**
Field / kHz	60 (^{1}H) / 45 (^{15}N)	60 (^{1}H) / 45 (^{15}N)	60 (^{1}H) / 42 (^{13}C)	60 (^{1}H) / 45 (^{15}N)
Shape	Tangent ^{1}H	Tangent ^{1}H	Tangent ^{1}H	Tangent ^{1}H
Carrier / ppm	120 (^{15}N)	120 (^{15}N)	59.3 (^{13}C)	120 (^{15}N)
Time / ms	1.0	0.8	0.5	1.0
**Transfer 2**	**NCO-CP**	**NCA-CP**	**CAN-CP**	**NCO-CP**
Field / kHz	21.1 (^{15}N) / 6.0 (^{13}C)	10.6 (^{15}N) / 6.0 (^{13}C)	6 (^{13}C) / 10.6 (^{15}N)	21.1 (^{15}N) / 6.0 (^{13}C)
Shape	Tangent ^{13}C	Tangent ^{13}C	Tangent ^{13}C	Tangent ^{13}C
Carrier / ppm	177.1 (^{13}C)	59.3 (^{13}C)	59.3 (^{13}C)	177.1 (^{13}C)
Time / ms	6.0	8.5	8.0	6.0
**Transfer 3**	-	-	**NCO-CP**	**Mod. band-selective CP**
Field / kHz	-	-	21.5 (^{15}N) / 6.0 (^{13}C)	8.1 (^{13}C)
Shape	-	-	Tangent ^{13}C	Tangent ^{13}C
Carrier / ppm	-	-	177.1 (^{13}C)	177.1 (^{13}C)
Time / ms	-	-	5.0	5.0
**Transfer 4**	-	-	**Mod. band-selective CP**	**DREAM**
Field / kHz	-	-	8.1 (^{13}C)	7.3 (^{13}C)
Shape	-	-	Tangent ^{13}C	Tangent ^{13}C
Carrier / ppm	-	-	177.1 (^{13}C)	56.0 (^{13}C)
Time / ms	-	-	5.3	1.75
Window function	QSINE SSB 3.0	QSINE SSB 3.0	QSINE SSB 2.5	QSINE SSB 2.5

Sample prep [a)]	h.	h.	h.	h.

[a)] Expression protocols in SI section listed for a. – k.

	CCC	hNH	hCH
MAS frequency / kHz	17	100	100
Field / T	20	20	20
Number of scans	8	64	40
t1 increment	158	300	780
Sweep width (t1) / ppm	70.0	120.0	180.0
Acquisition time (t1) / ms	5.3	14.5	10.1
t2 increments	156	2048	2048
Sweep width (t2) / ppm	70.0	46.7	46.7
Acquisition time (t2) / ms	5.2	25.8	25.8
t3 increments	2816	-	-
Sweep width (t3) / ppm	467.7	-	-
Acquisition time (t3) / ms	14.1	-	-
^{1}H decoupling / kHz	SPINAL64 / 90	swfTPPM / 10.5	swfTPPM / 10.1
^{15}N decoupling / kHz	-	WALTZ64 / 5.5	-
^{13}C decoupling / kHz	-	-	WALTZ64 / 5.4
Water suppression / kHz	-	MISS. 120 ms / 21.1	MISS. 120 ms / 20.4
Interscan delay / s	2.5	1.2	0.9
Experiment time	5 d 22 h	7 h	9 h
**Transfer 1**	**HC-CP**	**HN-CP**	**HC-CP**
Field / kHz	60 (^{1}H) / 42 (^{13}C)	77.8 (^{1}H) / 16.4 (^{15}N)	76.6 (^{1}H) / 16.5 (^{13}C)
Shape	Tangent ^{1}H	Tangent ^{1}H	Tangent ^{1}H
Carrier / ppm	98.6 (^{13}C)	117.5 (^{15}N)	56.0 (^{13}C)
Time / ms	0.5	0.9	0.6
**Transfer 2**	**DREAM**	**NH-CP**	**CH-CP**
Field / kHz	7.5 (^{13}C)	16.4 (^{15}N) / 77.8 (^{1}H)	16.5 (^{13}C) / 76.6 (^{1}H)
Shape	Tangent ^{13}C	Tangent ^{1}H	Tangent ^{1}H
Carrier / ppm	56 (^{13}C)	4.8 (^{1}H)	4.8 (^{1}H)
Time / ms	2.0	0.9	0.6
**Transfer 3**	**DARR**	-	-
Field / kHz	17 (^{1}H)	-	-
Shape	-	-	-
Carrier / ppm	98.6 (^{13}C)	-	-
Time / ms	70.0	-	-
Window function	QSINE SSB 2.5	QSINE SSB 3.0	QSINE SSB 3.0
TDeff		TD2eff 1024	TD2eff 1024
Sample prep [a)]	h.	h.	h.

[a)] Expression protocols in SI section listed for a. – k.

	hCANH	hNCAH	hNcoCAH	hCAcoNH
MAS frequency / kHz	100	100	100	100
Field / T	20	20	20	20
Number of scans	56	60	88	84
t1 increment	104	44	48	104
Sweep width (t1) / ppm	32.0	38.0	38.0	32.0
Acquisition time (t1) / ms	7.6	6.7	7.3	7.6
t2 increments	46	96	96	46
Sweep width (t2) / ppm	38.0	32.0	32.0	38.0
Acquisition time (t2) / ms	7.0	7.0	7.0	7.0
t3 increments	2048	2048	2048	3072
Sweep width (t3) / ppm	46.7	46.7	46.7	46.7
Acquisition time (t3) / ms	25.8	25.8	25.8	38.7
^{1}H decoupling / kHz	swfTPPM / 10.5	swfTPPM / 10.2	swfTPPM / 10.2	swfTPPM / 10.4
^{15}N decoupling / kHz	WALTZ64 / 5.5	WALTZ64 / 5.8	WALTZ64 / 5.0	WALTZ64 / 5.7
^{13}C decoupling / kHz	WALTZ64 / 5.0	WALTZ64 / 5.4	WALTZ64 / 4.9	WALTZ64 / 5.6
Water suppression / kHz	MISS. 120 ms / 21.0	MISS. 120 ms / 21.0	MISS. 120 ms / 20.4	MISS. 120 ms / 20.7
Interscan delay / s	1.0	1.0	1.0	1.0
Experiment time	3 d 16 h	3 d 17 h	5 d 14 h	6 d 16 h
**Transfer 1**	**HC-CP**	**HN-CP**	**HN-CP**	**HC-CP**
Field / kHz	76.4 (^{1}H) / 16.7 (^{13}C)	76.8 (^{1}H) / 16.8 (^{15}N)	77.9 (^{1}H) / 16.1 (^{15}N)	75.5 (^{1}H) / 16.9 (^{13}C)
Shape	Tangent ^{1}H	Tangent ^{1}H	Tangent ^{1}H	Tangent ^{1}H
Carrier / ppm	56.0 (^{13}C)	117.5 (^{15}N)	117.5 (^{15}N)	55.1 (^{13}C)
Time / ms	0.55	1.0	0.9	0.5
**Transfer 2**	**CN-CP**	**NC-CP**	**NCO-CP**	**DREAM**
Field / kHz	64.5 (^{13}C) / 33.8 (^{15}N)	33.8 (^{15}N) / 63.6 (^{13}C)	32.7 (^{15}N) / 63.6 (^{13}C)	45.1 (^{13}C)
Shape	Tangent ^{13}C	Tangent ^{13}C	Tangent ^{13}C	Tangent ^{13}C
Carrier / ppm	117.5	56.0 (^{13}C)	222.1 (^{13}C)	184.1 (^{13}C)
Time / ms	13.5	16.0	13.0	7.0
**Transfer 3**	**NH-CP**	**CH-CP**	**DREAM**	**CN-CP**
Field / kHz	16.6 (^{15}N) / 78.6 (^{1}H)	16.6 (^{13}C) / 75.5 (^{1}H)	47.2 (^{13}C)	65.3 (^{13}C) / 33.7 (^{15}N)
Shape	Tangent ^{1}H	Tangent ^{1}H	Tangent ^{13}C	Tangent ^{13}C
Carrier / ppm	4.8 (^{1}H)	4.8 (^{1}H)	156.1 (^{13}C)	117.5
Time / ms	1.0	0.5	9.0	14.5
**Transfer 4**	-	-	**CAH-CP**	**NH-CP**
Field / kHz	-	-	16.7 (^{13}C) / 73.3 (^{1}H)	16.6 (^{15}N) / 76.6 (^{1}H)

Shape	-	-	Tangent ^{1}H	Tangent ^{1}H
Carrier / ppm	-	-	4.8 (^{1}H)	4.8 (^{1}H)
Time / ms	-	-	0.5	1.1
Window function	QSINE SSB 3.0	QSINE SSB 3.0	QSINE SSB 2.5	QSINE SSB 2.7
TDeff	TD3eff 1024	TD3eff 1024	TD3eff 1024	TD3eff 1024
Sample prep [a)]	h.	h.	h.	h.

[a)] Expression protocols in SI section listed for a. – k.

Table S 4 Validation of cryo-EM structures

Parameter [a)]	ACNDV capsid pH 7.5	ACNDV capsid pH 5.5
Chains	180	180
Residues [b)]	122 per chain	122 per chain
Bond lengths RMSD (Å) [c)]	0.008	0.005
Bond angles RMSD (°) [c)]	1.060	1.144
MolProbity score [c)]	0.89	1.24
Clash score [c)]	1.47	2.04
Ramachandran Outliers (%) [c)]	0.00	0.00
Ramachandran Allowed (%) [c)]	1.98	3.95
Ramachandran Favored (%) [c)]	98.02	96.05
Rotamer outliers (%) [c)]	0.00	0.90
CaBLAM outliers (%) [c)]	0.00	1.17
ADP (B-factor) mean (Å^2)	68.73	55.67
d FSC model vs map masked 0.143/0.5 (Å) [d)]	3.5/3.9	3.8/n.a.
CC mask/peaks/volume [d)]	0.76/0.67/0.75	0.69/0.51/0.66

[a] Comprehensive validation for cryo-EM implemented in *Phenix* [1]. [b] Residues 0-2 and 66-75 were removed after structure building and refinement which leaves 122 residues. [c] MolProbity [2-4] available in *Phenix*. [d] Mtriage [5] available in *Phenix*.

Table S 5: Connectivity in the ACNDV spike region

Residue	NMR connections	NMR assignment	Cryo-EM map density
65Asp	64Leu, 66Leu	confirmed	visible
66Leu	65Asp, 67Ala	confirmed	invisible
67Ala	66Leu, 68Gly	confirmed	invisible
68Gly	67Ala	confirmed	invisible
69Lys	-	tentative	invisible
70Ala/75Ala	-	tentative	invisible
71Thr	-	tentative	invisible
72Ser	-	tentative	invisible

73Asn	-	tentative	invisible
74Glu	-	tentative	invisible
75Ala/70Ala	-	tentative	visible
76Lys	77Pro	confirmed	visible

Table S 6: Residues and concerned atoms for which peak splitting or peak broadening was observed in spectra of ACNDV capsids at pH 7.5.

Residue	Atom	Spectra
3Phe	H (3peaks)	hNH
4Ile	H (broadened)	hNH
14Lys	H (broadened)	hCANH & also hNH slightly broadened
26Pro	CB (broadened)	NcoCACB
29Val	CB (broadened); H (broadened)	NcoCACB but not DARR and NCACB; hCANH
31Lys	Cg (broadened)	NCACX & maybe also in DARR
46Trp	CB (broadened); Cg (broadened); He1 (too many peaks)	DARR; DARR; hNH
94Trp	CB (almost two peaks); Cg (broadened); Cd1 (broadened but bad S/N); He1 (too many peaks)	NcoCACB; DARR; DARR; hNH
97Ile	HA (broadened)	hNCAH
111Val	HA (two peaks); H (two peaks)	hNCAH; hCANH
117 Trp	CB (broadened); Cg (broadened); He1 (too many peaks)	DARR; DARR; hNH
120Thr	Cg (broadened)	NCACX & DARR
123Ala	CO (broadened)	NCACX

Table S 7: T_2’ relaxation

Parameter	ACNDV / ms (sd of the fit)	HBV / ms
T_2’	2.282(9) [a)]	2.2 [6] [b)]

[a)] Bulk relaxation of hNH CP T_2’ experiment with 12 data points, 0.7 mm rotor at 100 kHz MAS, 850 MHz spectrometer, delays from 1 µs to 3.25 ms, integration over whole H dimension (0-14 ppm). The T_1T_2 relaxation module from Topspin was used for integration and fitting. [b)] 0.7 mm rotor at 100 kHz MAS, 850 MHz spectrometer, fully protonated full length protein sample.

Table S 8: Cryo-EM measurements

	pH 7.5	pH 7.5 asu	pH 5.5	pH 7.5 dia	pH 5.5 dia	pH shift dia
microscope	Titan Krios		Titan Krios	Titan Krios	Titan Krios	Titan Krios
voltage (kV)	300		300	300	300	300
magnification	130'000 x		166'600 x	129'000 x	129'000 x	129'000 x
pixel size Å/pixel	1.07		0.845	1.087	1.087	1.087
dose $e^-/\text{Å}^2$	76		55	77	77	77
defocus (μm)	-0.5 to -2		-0.2 to -2	-1 to -2.6	-1 to -2.6	-1 to -2.6
detector	K2		Falcon III	Falcon III	Falcon III	Falcon III
slith width (eV)	20					
detector mode	counting		linear	linear	linear	linear
symm. imp.	I	C1 (asu)	I	I	I	I
micrographs	8'999	8'999	5'362	1'515	4'023	2'384
particles picked	375'654	375'654	215'225	146'077	346'287	257'179
particles (final)	70'868	1'895'898 (asu)	77'129	38'355	141'485	61'336
resolution (Å)	3.7	3.8	3.9	4.1	3.8	3.9

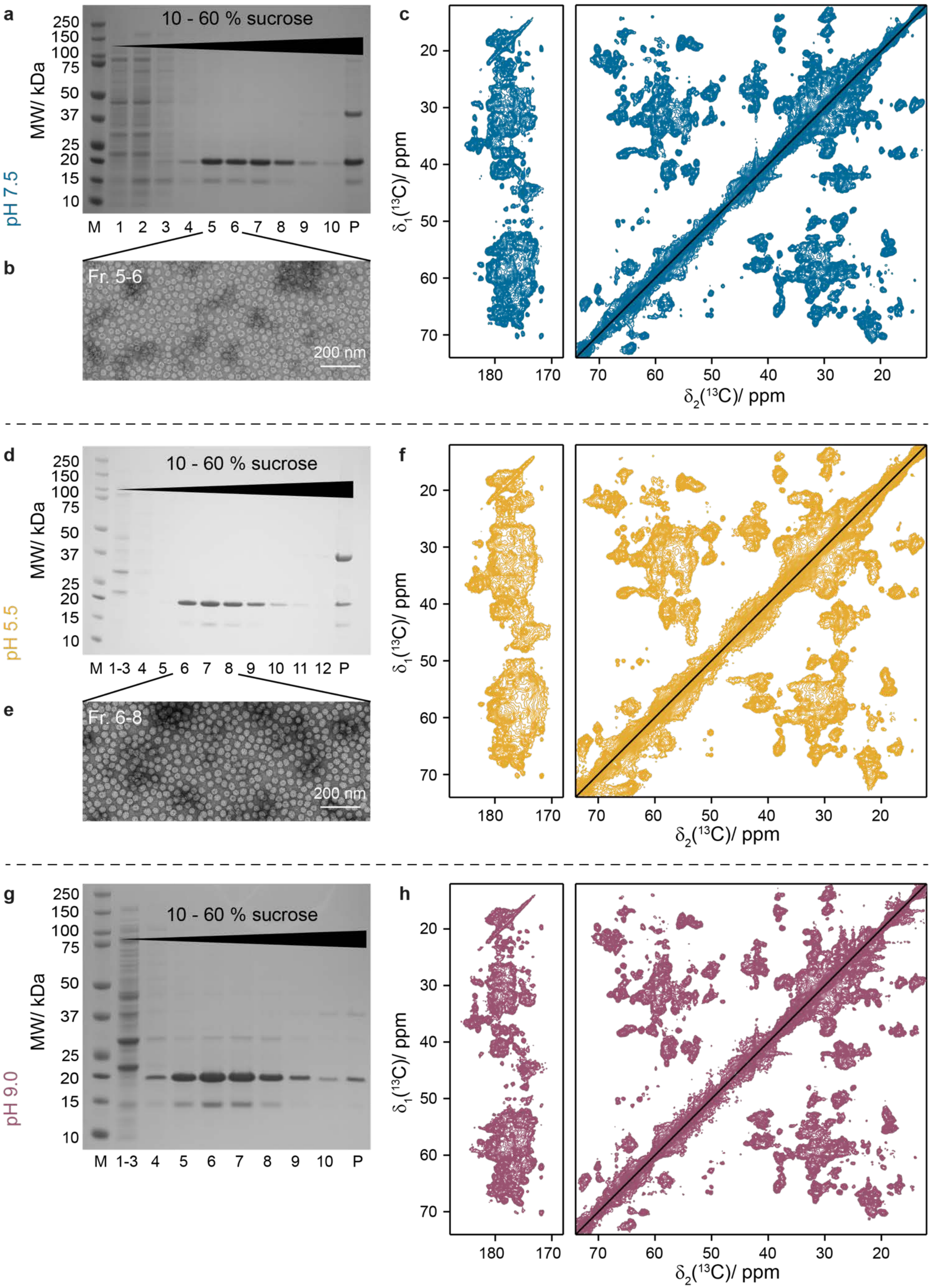
a
10 - 60 % sucrose
MW/ kDa
250
150
100
75
50
37
25
20
15
10
pH 7.5
M 1 2 3 4 5 6 7 8 9 10 P
b
Fr. 5-6
200 nm
c
δ1(13C)/ ppm
δ2(13C)/ ppm
d
10 - 60 % sucrose
pH 5.5
M 1-3 4 5 6 7 8 9 10 11 12 P
e
Fr. 6-8
200 nm
f
g
10 - 60 % sucrose
pH 9.0
M 1-3 4 5 6 7 8 9 10 P
h

Figure S 1: **Expression and DARR spectra. a, b, c)** ACNDV capsid protein in TRIS buffer at pH 7.5. **d**, **e**, **f)** ACNDV capsid protein in acetate buffer at pH 5.5. The protein was expressed and purified at pH 7.5 and subjected to the pH 5.5 buffer by dialysis after purification. **(g**, **h)** ACNDV capsid protein in TRIS buffer at pH 9.0. The expression and purification of the protein was performed at pH 7.5. The sample was subjected to the higher pH by stepwise washing with pH 9.0 buffer in a 100 kDa cutoff concentrator. **a)** SDS-PAGE of fractions of the sucrose gradient. The ACNDV capsid protein has a molecular weight (MW) of 19.8 kDa and sediments in the fractions containing 20-40 % sucrose (Fr. 5 -8). Additionally, the protein is found in the pellet of the sucrose gradient centrifugation. A band at 15 kDa remains in the purified fractions. The 15-kDa protein cannot be separated from the ACNDV capsid protein by centrifugation in a 100 kDa cutoff concentrator or by size exclusion chromatography (Sephacryl S-400). The identity of the 15-kDa contamination was not further researched. Presumably, it comprises a C-terminal cleaved version of the capsid protein. **b)** Negative stain image obtained by TEM displaying the particles before filling the NMR rotor. Fractions 5-6 from the sucrose gradient were concentrated together with additional protein material of which the sucrose gradient sedimentation profile is not shown. **c)** Carbon-carbon DARR correlation spectrum of the ACNDV pH 7.5 capsid protein sample shown in the micrograph. The protein was sedimented into the 3.2 mm rotor by centrifugation. To obtain carbon-detected spectra the rotor was spun at 17 kHz MAS at an 850 MHz spectrometer. The DARR mixing time was set to 20 ms. **d)** SDS-PAGE of fractions of the sucrose gradient of the ACNDV capsid protein for the NMR spectrum at pH 5.5. Since expression was performed at pH 7.5, the sedimentation behavior in the sucrose gradient is identical to the gradient shown in panel A. The difference in fraction numbers comes from different fraction sizes: 3mL per fraction compared to 3.5 mL per fraction in panel A. **e)** Negative stain image obtained by TEM of the particles after dialysis into acetate buffer at pH 5.5. Fractions 6-8 of the sucrose gradient were combined for NMR rotor filling. **f)** Carbon- carbon DARR correlation spectrum of the ACNDV capsid protein in pH 5.5 buffer. Regions with broad unresolved signal could originate from denatured protein (see Figure S 2c and d). **g)** SDS-PAGE displaying the fractions of the sucrose gradient of the ACNDV capsid protein at pH 9.0. Since expression was performed at pH 7.5, the sedimentation behavior in the sucrose gradient is identical to the gradient shown in panel A. Fractions 5-7 were filled into a 3.2 mm NMR rotor. **h)** DARR spectrum of ACNDV capsids at pH 9.0. The experiment was performed at 17 kHz MAS at a 850 MHz spectrometer and the DARR mixing time was set to 20 ms. The spectra at pH 7.5 and pH 9.0 look the same (see superposition in Figure S 2b) except for the pH 9.0 spectrum displaying the missing lysine feature that is further discussed in Figure S 22. This feature supposedly has nothing to do with the buffer pH.

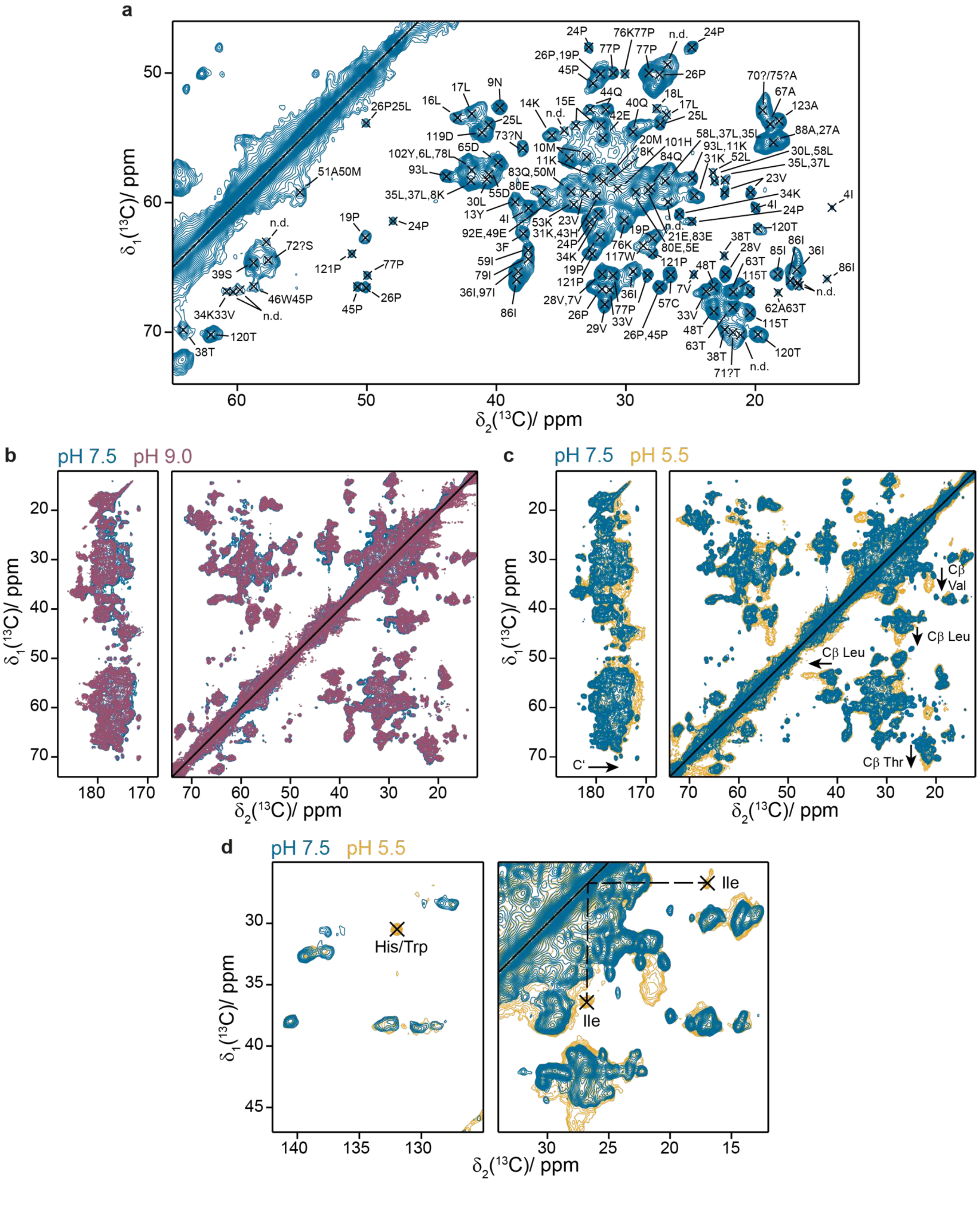

a
δ1(13C)/ ppm
δ2(13C)/ ppm
b
pH 7.5
pH 9.0
δ1(13C)/ ppm
δ2(13C)/ ppm
c
pH 7.5
pH 5.5
C'
Cβ Val
Cβ Leu
Cβ Leu
Cβ Thr
δ1(13C)/ ppm
δ2(13C)/ ppm
d
pH 7.5
pH 5.5
His/Trp
Ile
Ile
δ1(13C)/ ppm
δ2(13C)/ ppm

Figure S 2: **NMR assignment at pH 5.5 / pH 9.0. a)** Assigned DARR spectrum of ACNDV capsids at pH 7.5. Not assigned peaks are denoted with "n.d.". A question mark (?) denotes tentatively assigned peaks from the capsid spike (see Figure 4). **b)** Overlay of DARR spectra of ACNDV capsids at pH 7.5 (blue) and ACNDV capsids at pH 9.0 (purple). The two spectra are shown separately in Figure S 1c (pH 7.5) and h (pH 9.0). The two spectra are very similar except for the weakened lysines in the pH 9.0 spectrum (see Figure S 22). **c)** Overlay of DARR spectra of ACNDV capsids at pH 7.5 (blue) and at pH 5.5 (yellow). The two spectra are shown separately in Figure S 1c (pH 7.5) and F (pH 5.5). The shift of C' resonances and Cβ resonances of valines, leucines, and threonines are indicated with arrows. The changes are indicative of β-strand formation.**d)** Zoom into overlay (panel c) comparing DARR spectra at pH 7.5 (blue) and pH 5.5 (yellow). The additional broad unresolved signals in the pH 5.5 are ascribed to denatured protein. Protein samples that stayed in the NMR rotor for several months yields broad unresolved features (Figure S 4) similar to the spectrum at pH 5.5. Apart from the broad unresolved areas, the spectrum at pH 5.5 displays 3 additi2onal peaks that are not present in the spectrum at pH 7.5. Two peaks presumably belong to the same isoleucine spin system. The peak in the aromatic region may belong to a tryptophan or histidine based on the chemical shifts. It is not clear whether the additional peaks in the pH 5.5 spectrums are shifted peaks that are located in overlapping regions for the pH 7.5 sample or if these are newly appearing peaks. In the NMR spectra the main effect of lowering the pH is the faster denaturation of protein at pH 5.5 compared to pH 7.5.

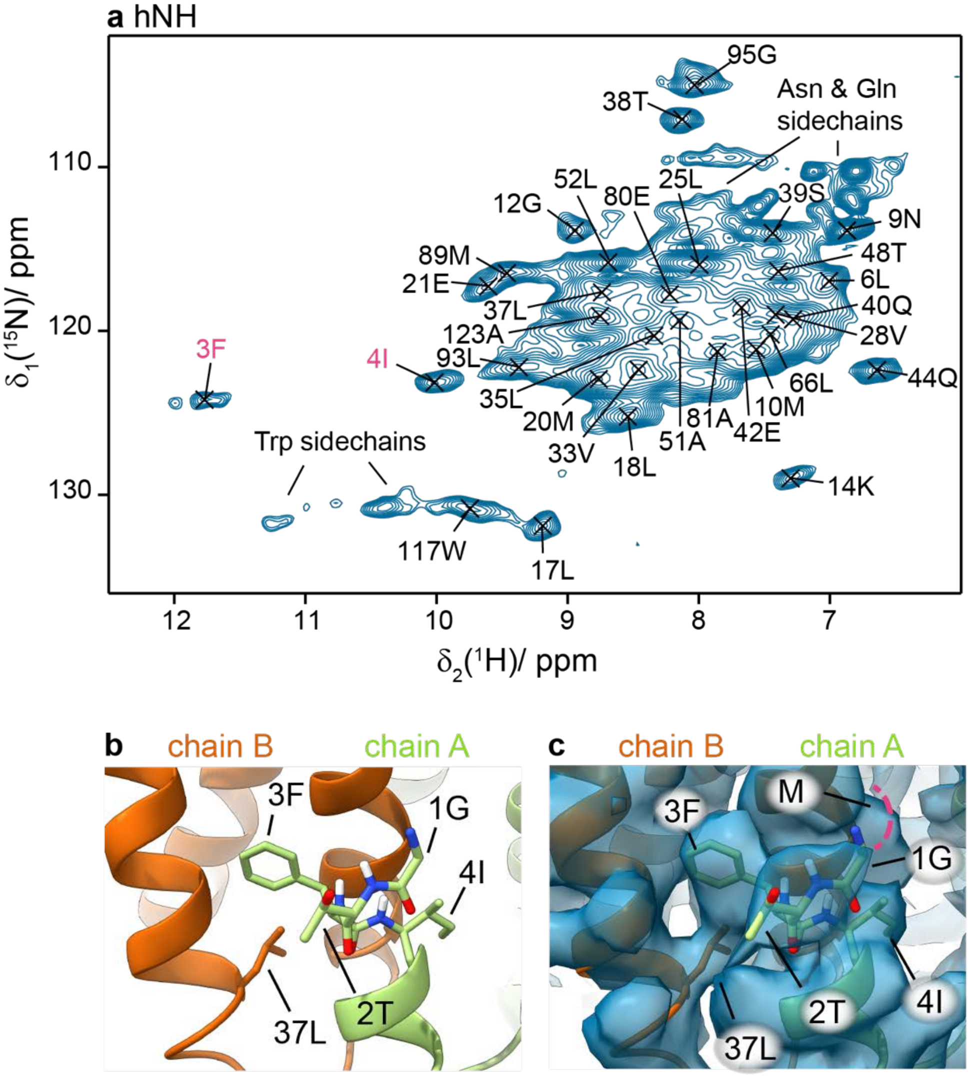

Figure S 3: **NMR hNH spectrum and N-terminus of ACNDV Cp. a)** Proton-detected hNH spectrum of ACNDV capsids at pH 7.5 recorded on an 850 MHz spectrometer. The MAS frequency was 100 kHz. Asn, Gln, and Trp sidechain regions are indicated but were not assigned. 3F and 4I are highlighted in pink. These peaks, especially, the 3F peak, have a high proton chemical shift. For comparison, the Biological Magnetic Resonance Data Bank (BMRB) calculates an average amide proton chemical shift of 8.3 ppm (standard deviation 0.7 ppm) for all deposited phenylalanine residues and a shift of 8.21 ppm (standard deviation 0.7 ppm) for isoleucines [7]. Such a high proton chemical shift as it was found for 3Phe is often caused by hydrogen bonding [8, 9]. A significant ring-current effect of the aromatic sidechain of 3Phe can be ruled out in our case. Moreover, the 3F peak is split into three subpeaks. **b)** Zoom into the ACNDV pH 7.5 cryo-EM structure at the N-terminus of chain A. 3F and 4I are the first resolved-residues of the protein sequence in the NMR spectra. **(C)** ACNDV capsid pH 7.5 and cryo-EM density map. Continuous density between 3F and 37L sidechains is visible. The unresolved methionine at the N-terminus is drawn as a pink line.

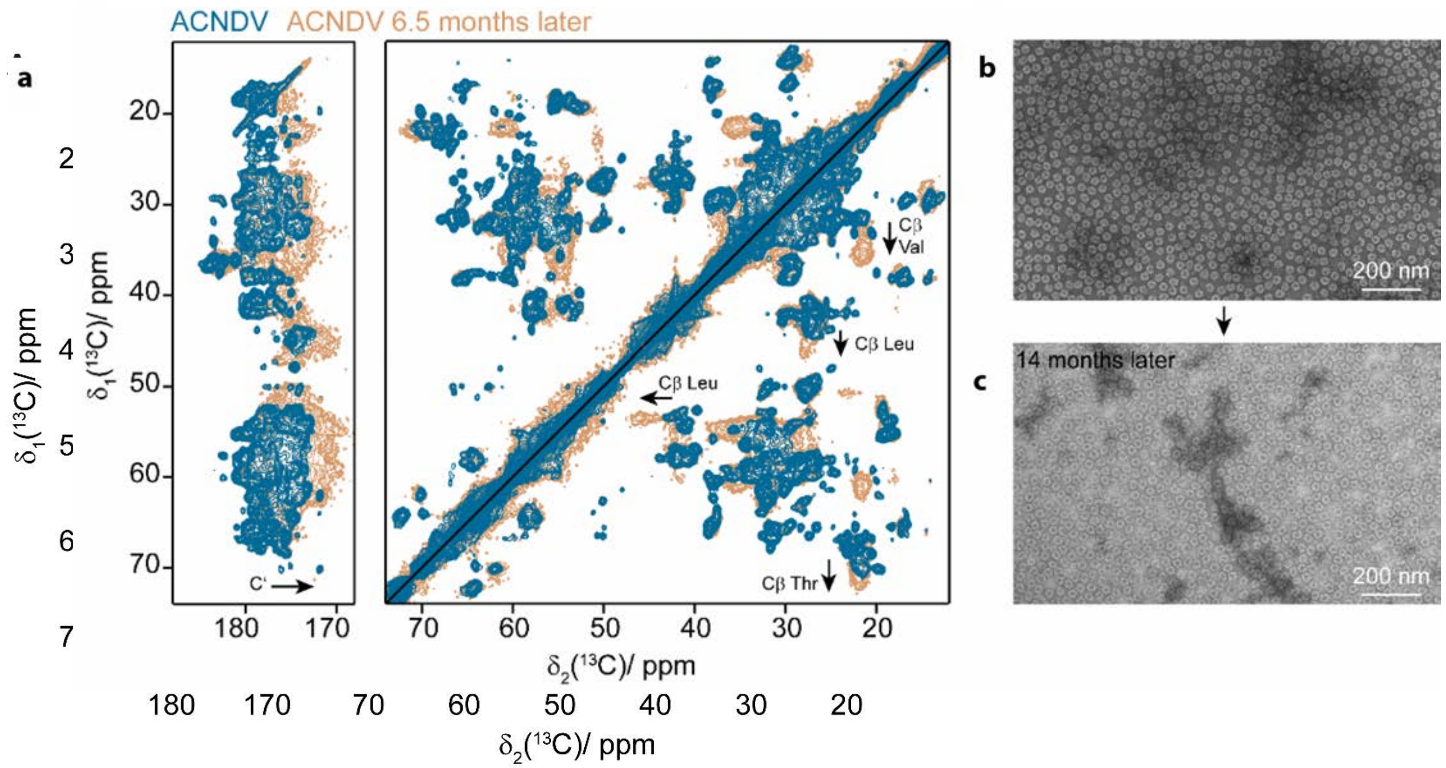

Figure S 4: **ACNDV Cp sample denaturation**. **a)** DARR spectrum of ACNDV capsid protein at pH 7.5 measured 2 months after NMR rotor filling (blue) superimposed onto the DARR spectrum of the same sample 6.5 months after rotor filling (light brown). Broad unresolved features appear after the sample staying a few months in the NMR rotor. These features indicate denaturation of the protein. This behavior was observed after a few months for all ACNDV protein samples. The shift of C' resonances and Cβ resonances of valines, leucines, and threonines are indicated with arrows. The changes are symptomatic for β-strand formation. The spectral fingerprints of protein that stayed in the NMR rotor for 6.5 months and the spectrum at pH 5.5 are highly similar (see Figure S 2C). **b**, **c)** ACNDV pH 7.5 capsid protein particles viewed by negative stain transmission electron microscopy. Notc: The samples are not identical with the NMR samples in panel A. The image in panel B displays the sample 5 months post expression and panel C the same sample 14 months after image B was recorded. The protein sample was stored in TRIS buffer at 4 °C. No obvious signs of denaturation could be detected in the transmission electron microscopy images. This implies that denaturation is accelerated when the sample stays in the NMR rotor.

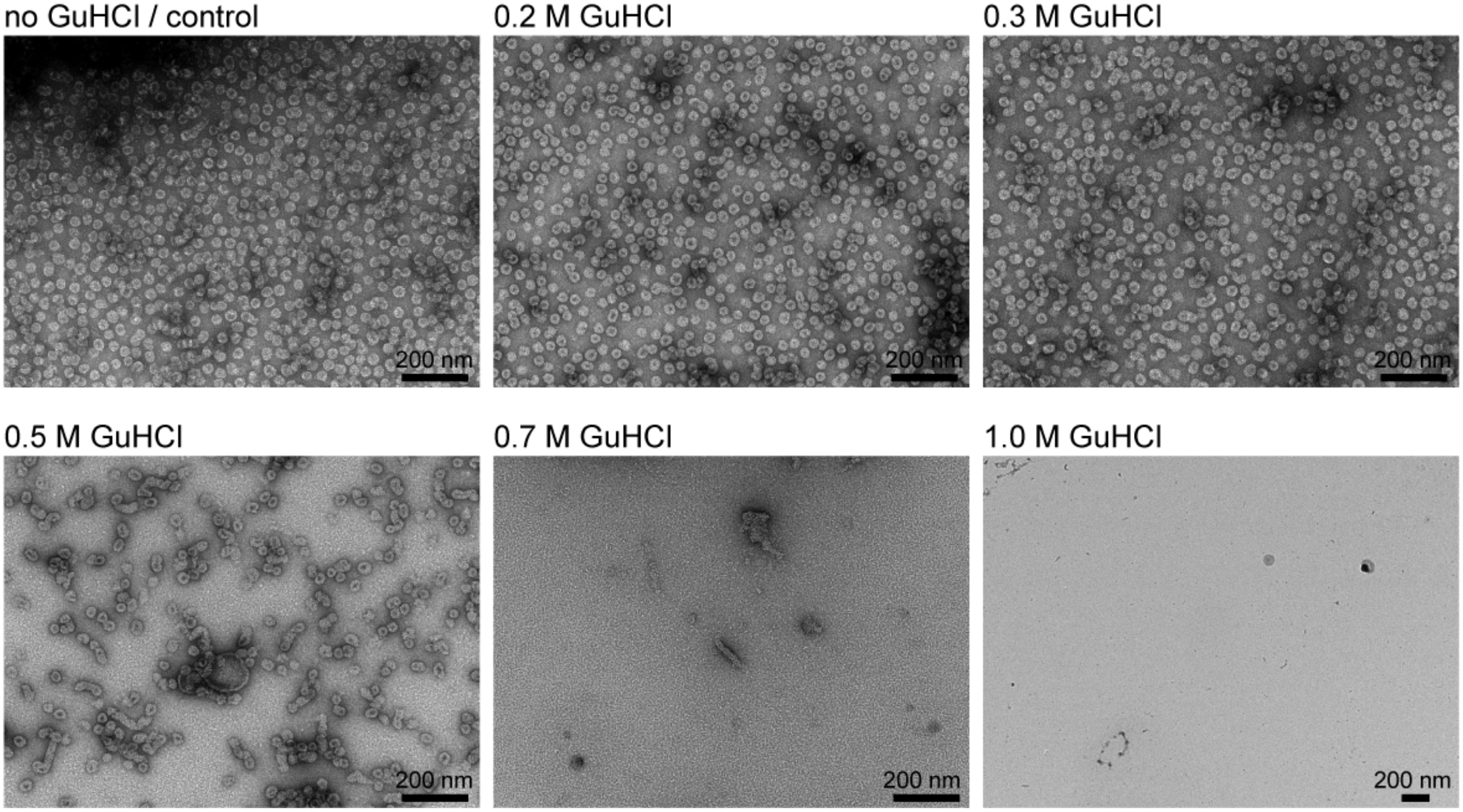

Figure S 5: **ACNDV capsids and GuHCl.** TEM images of ACNDV capsid samples treated with different concentrations of GuHCl.

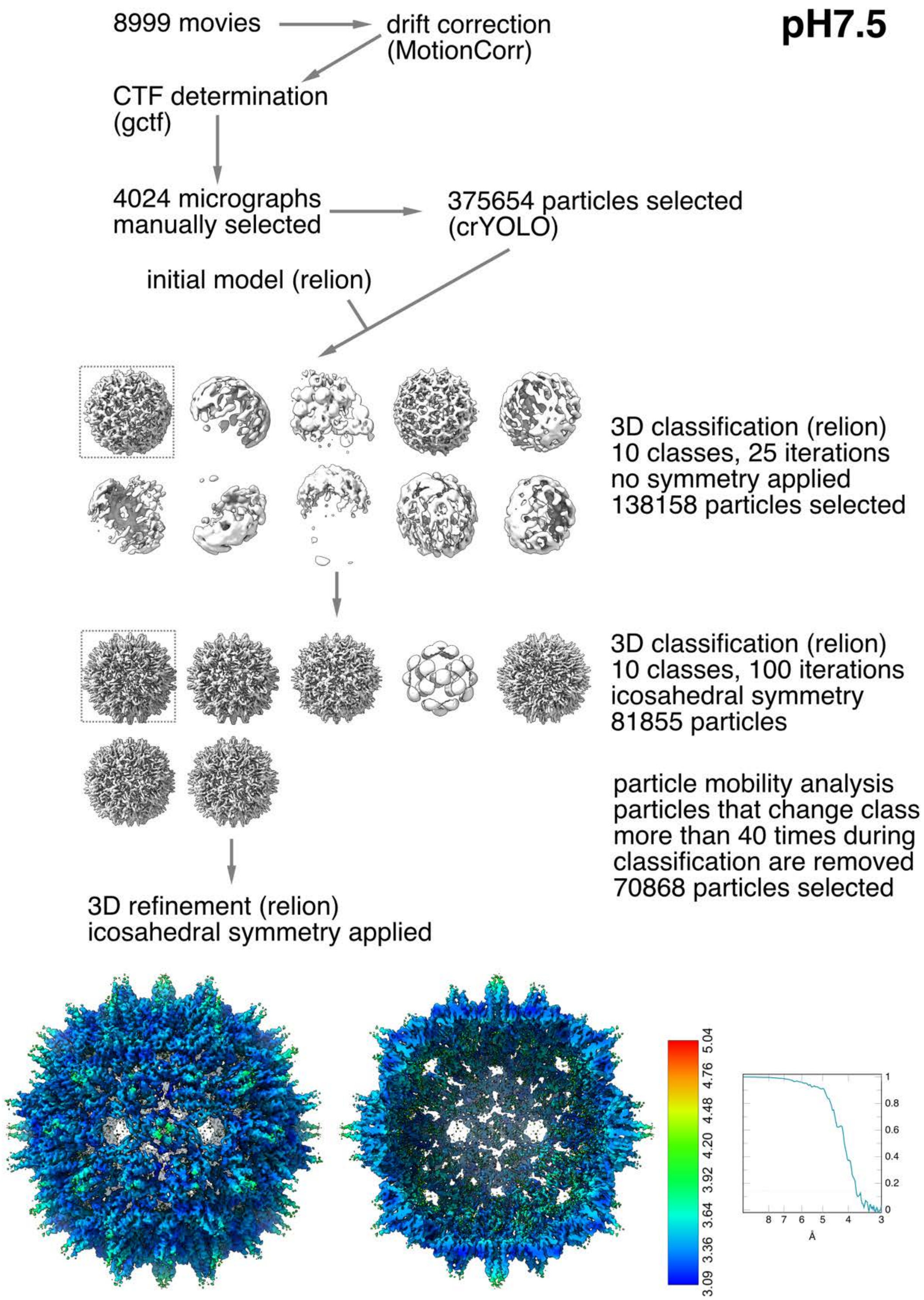

Figure S 6: **Cryo-EM image processing scheme** for the ACNDV capsid at pH 7.5 with resulting maps colored according to local resolution (obtained with *Phenix* [1]) and with corresponding FSC curve.

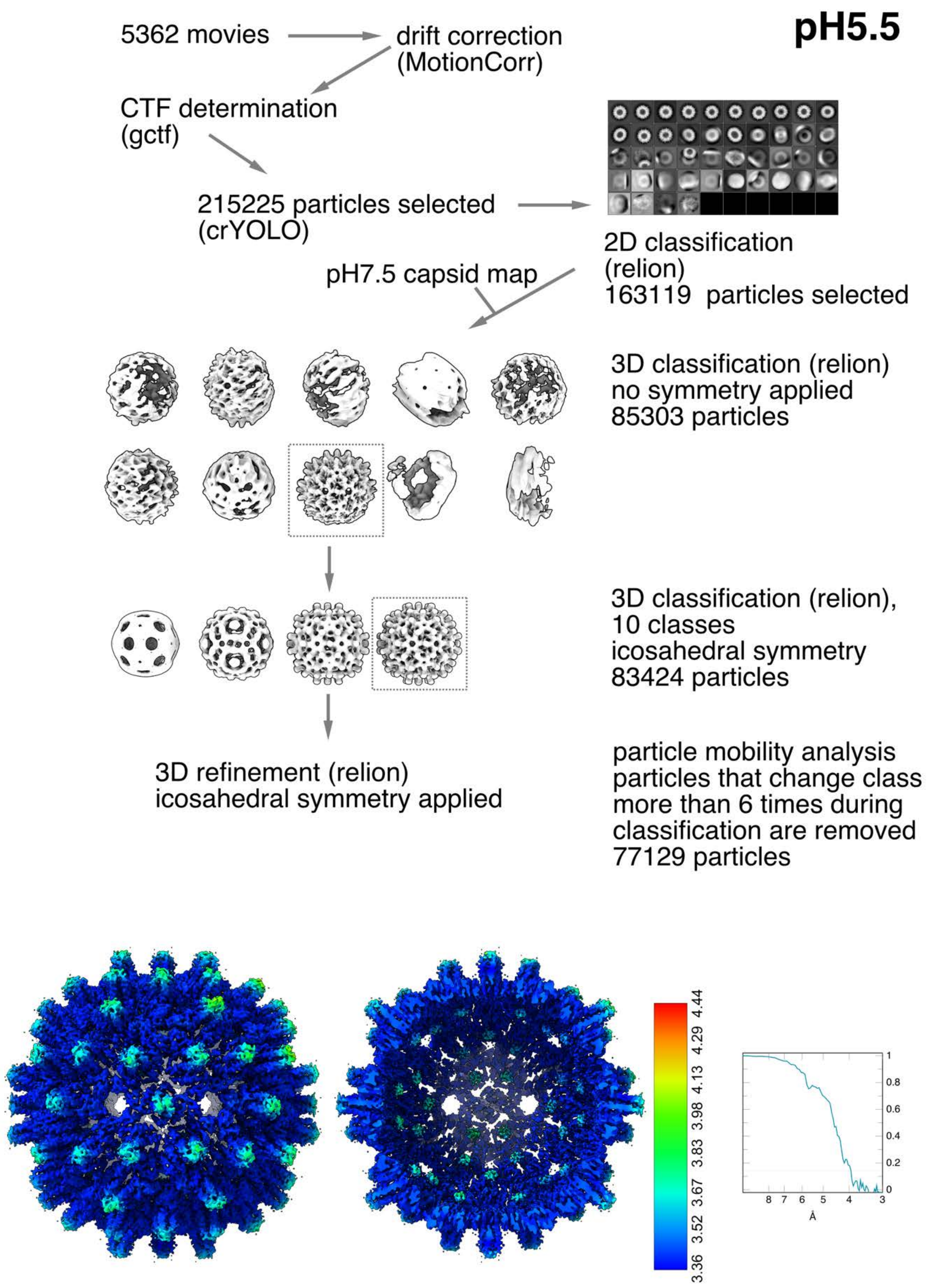

Figure S 7: **Cryo-EM image processing scheme** for the ACNDV capsid at pH 5.5 with resulting maps colored according to local resolution (obtained with *Phenix* [1]) and with corresponding FSC curve.

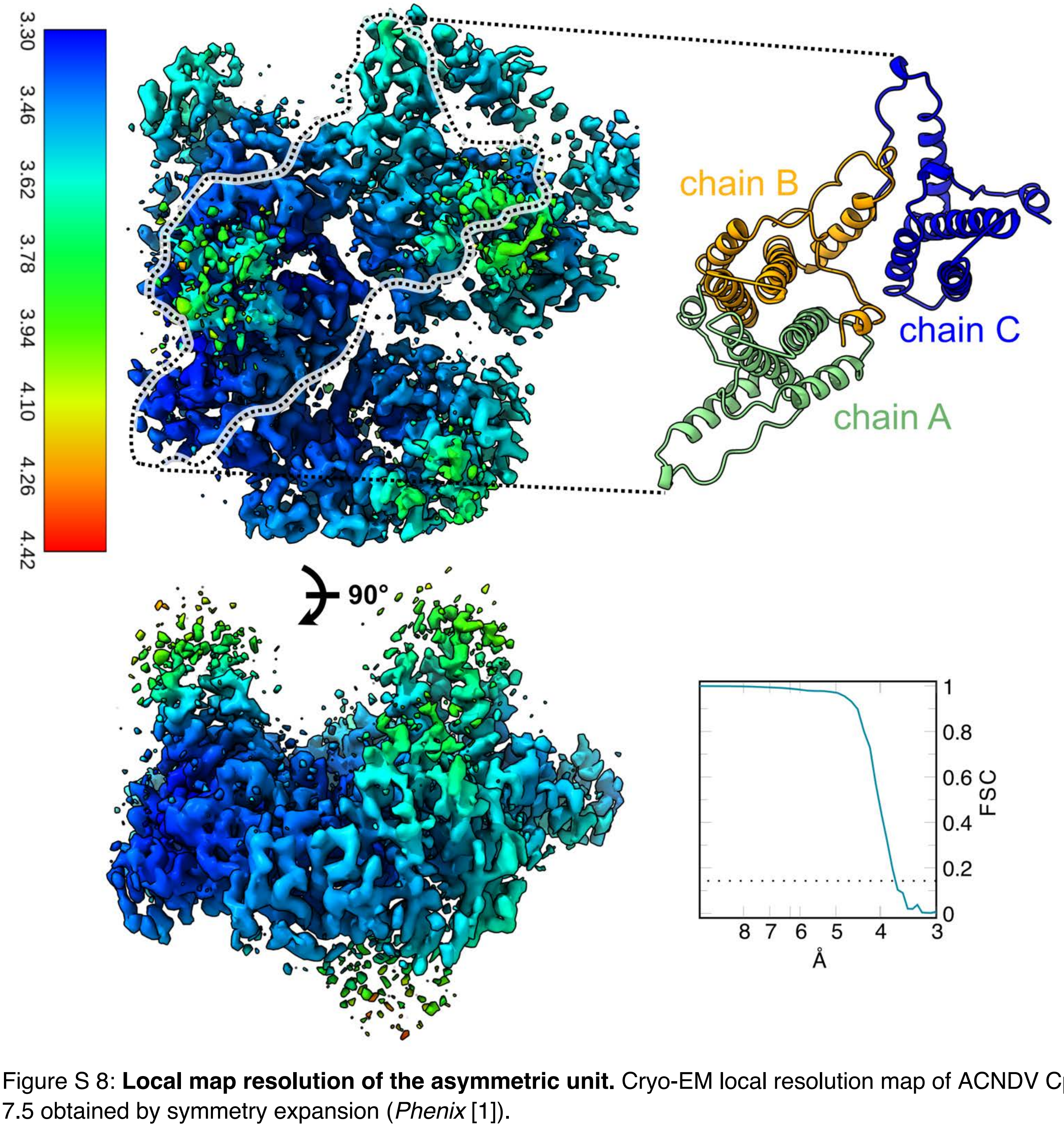

Figure S 8: **Local map resolution of the asymmetric unit.** Cryo-EM local resolution map of ACNDV Cp at pH 7.5 obtained by symmetry expansion (*Phenix* [1]).

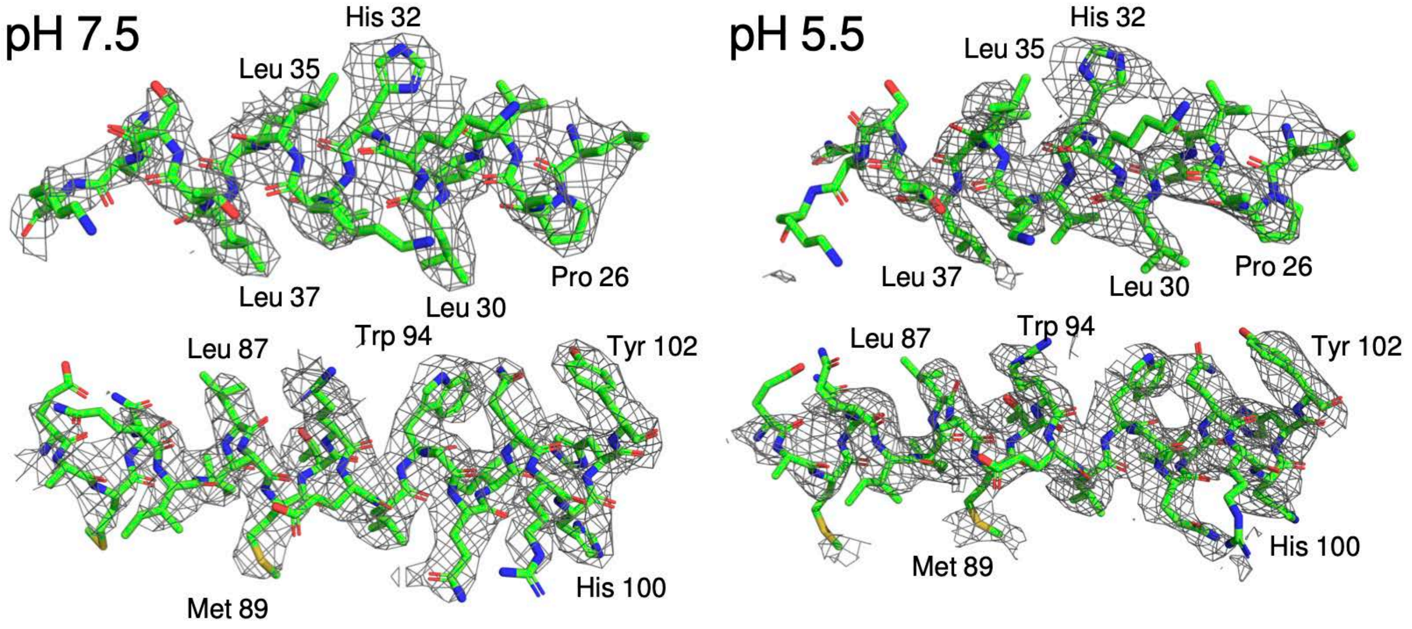

Figure S 9: **Fitted models and maps.** ACNDV Cp at pH 7.5 and at pH 5.5 showing residues 26-37 and 87-102.

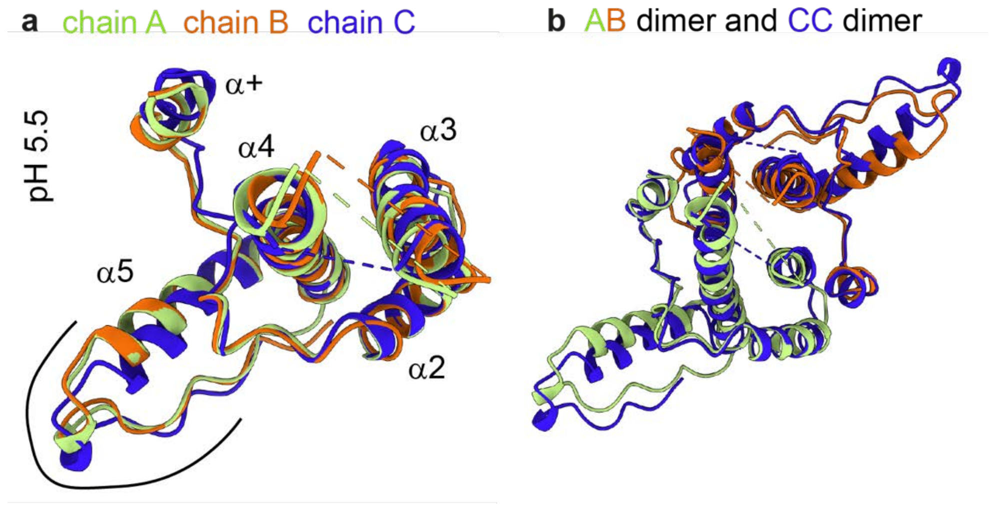

Figure S 10: **ACNDV Cp chains at pH 5.5 a)** ACNDV at pH 5.5 chains were superimposed by the matchmaker command implemented in ChimeraX [10-12] Black lines delineate the hand region of the capsid protein. Dotted lines indicate the protein chain connectivity. **b)** Superposition of the AB and CC dimer at pH 5.5.

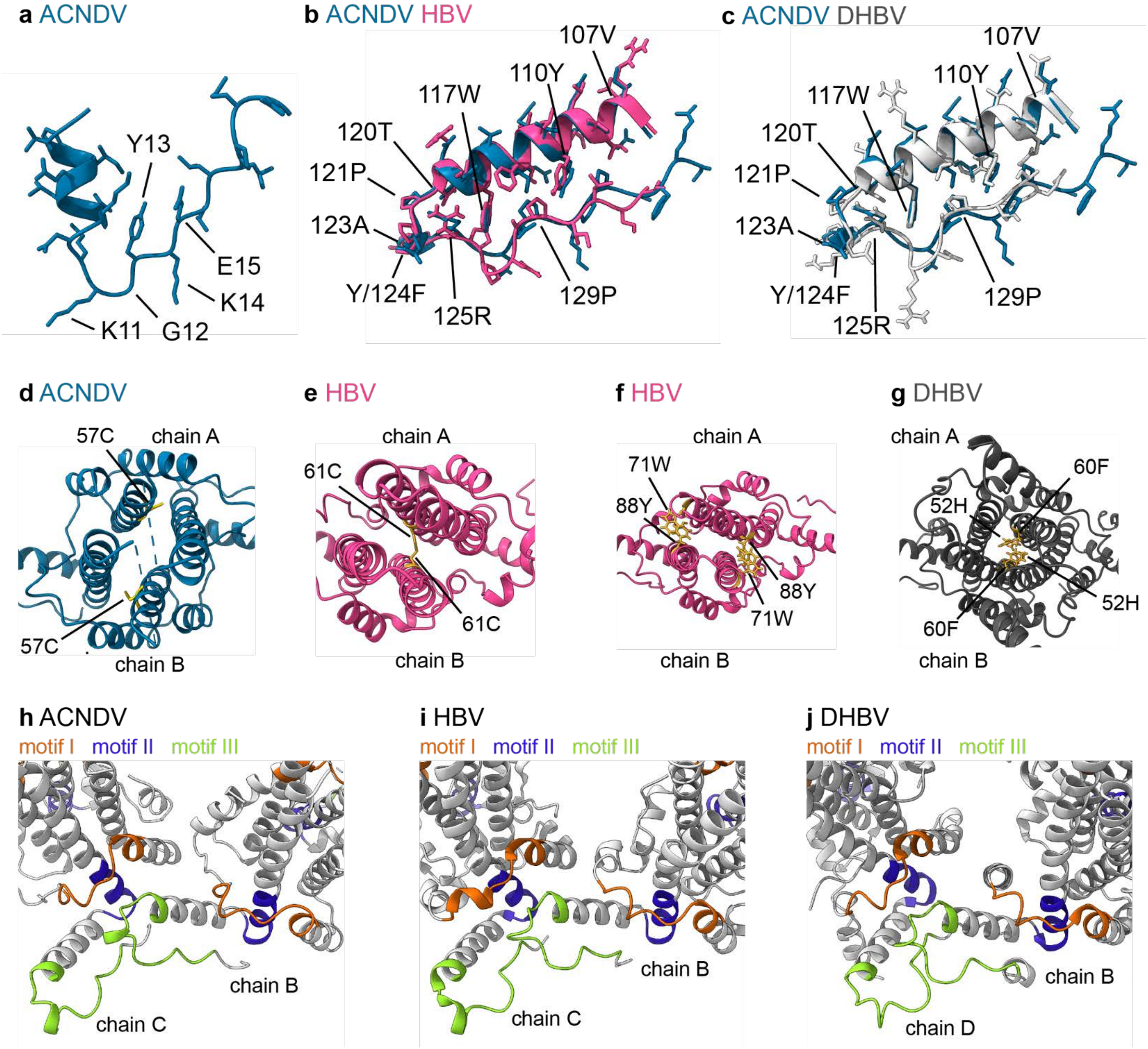

Figure S 11: **Structural details of ACNDV, HBV, and DHBV capsid protein.** The structures were superimposed with the matchmaker command of ChimeraX [10, 12]. **a)** N-terminus of ACNDV Cp showing sidechains pointing in alternate directions. **b)** Hand region of chain A of ACNDV (blue) and HBV (pink, pdb 6ui6 [13]) capsid protein with conserved amino acids: V155/V107, Y118/Y110, W125/W117, T128/T120, P129/P121, A131/A123, Y132/F124, R133/R125, and P138/P129 for HBV/ACNDV with the residue numbers of ACNDV Cp indicated in the image. **c)** Hand region of chain A of ACNDV (blue) and DHBV (gray, pdb 6ygh [14]) capsid protein with conserved amino acids: V161/V107, Y164/Y110, W171/W117, T174/T120, P175/P121, Y178/F124, R179/R125, P184/P129 for DHBV/ACNDV with residues number of ACNDV Cp shown in the image. **d)** Chain A and B of ACNDV capsid at pH 7.5. The cysteines are colored in yellow. They are far apart and no disulfide bond is formed. **e)** Chain A and B of HBV T=4 capsid (pdb 6htx) [15]. A disulfide bond can be formed across the spike. **f)** Chain A and B of HBV T=3 capsid (pdb 6ui6) [13].

Residues W71 and Y88 stabilize the helices. **g)** Chain A and B of DHBV T=4 capsid (pdb 6ygh) [14]. H52 and H52 interact across the spike helices of opposing monomers. The same holds true for the F60 residues. **h, i, j)** The structures of the three core motifs; motif I (orange), II (blue), and III (green) identified by [16], are highly similar in ACNDV, HBV (pdb 6ui6 [13]), and DHBV (pdb 6ygh [14]) capsids.

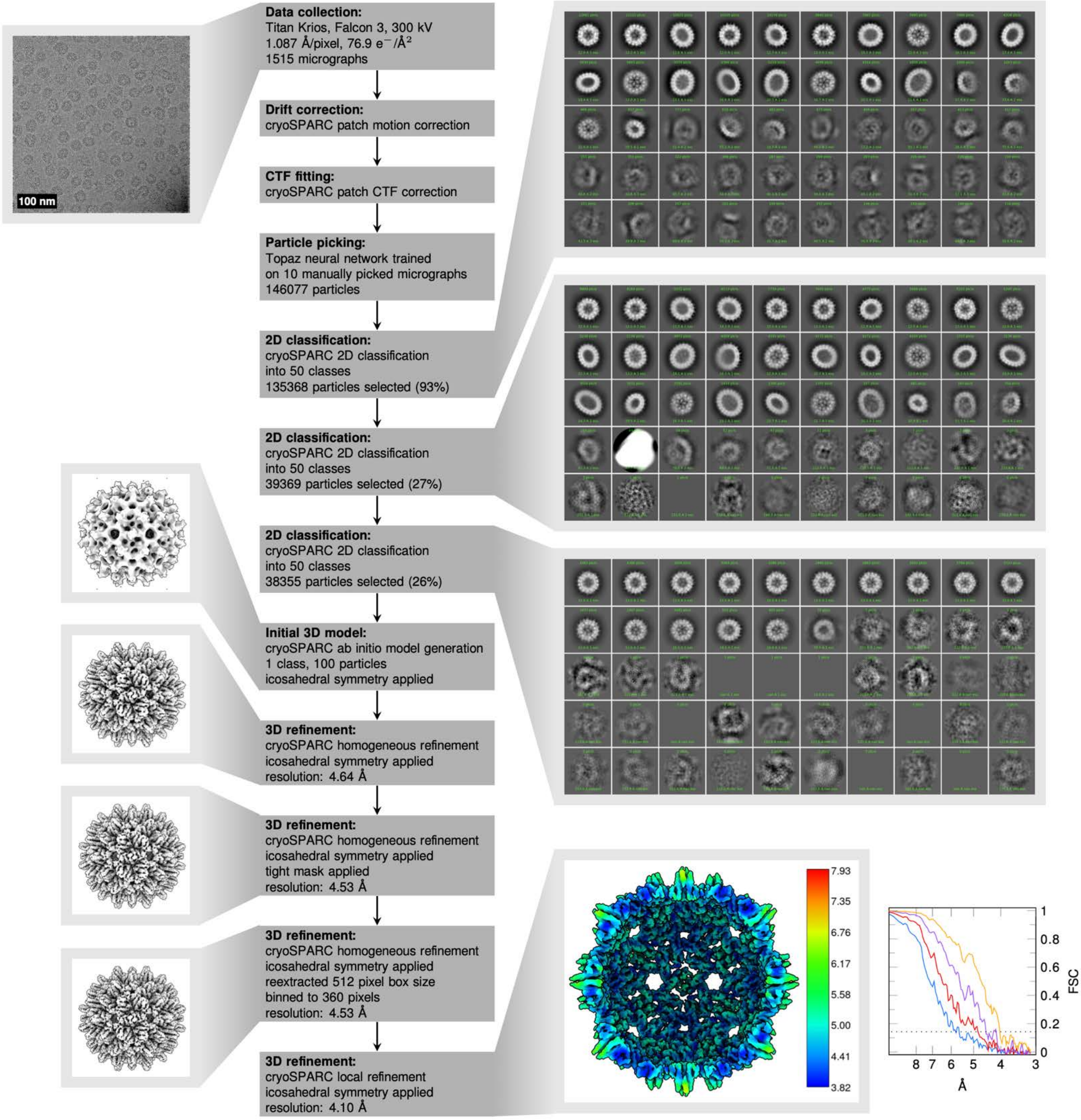

Figure S 12: **Cryo-EM image processing scheme** for the ACNDV capsid pH shift experiment. Processing scheme for ACNDV capsid at pH 7.5. The Fourier shell correlation is plotted for the map without masking (blue), with a spherical mask (red), with a loose mask (purple), and with a tight mask (orange).

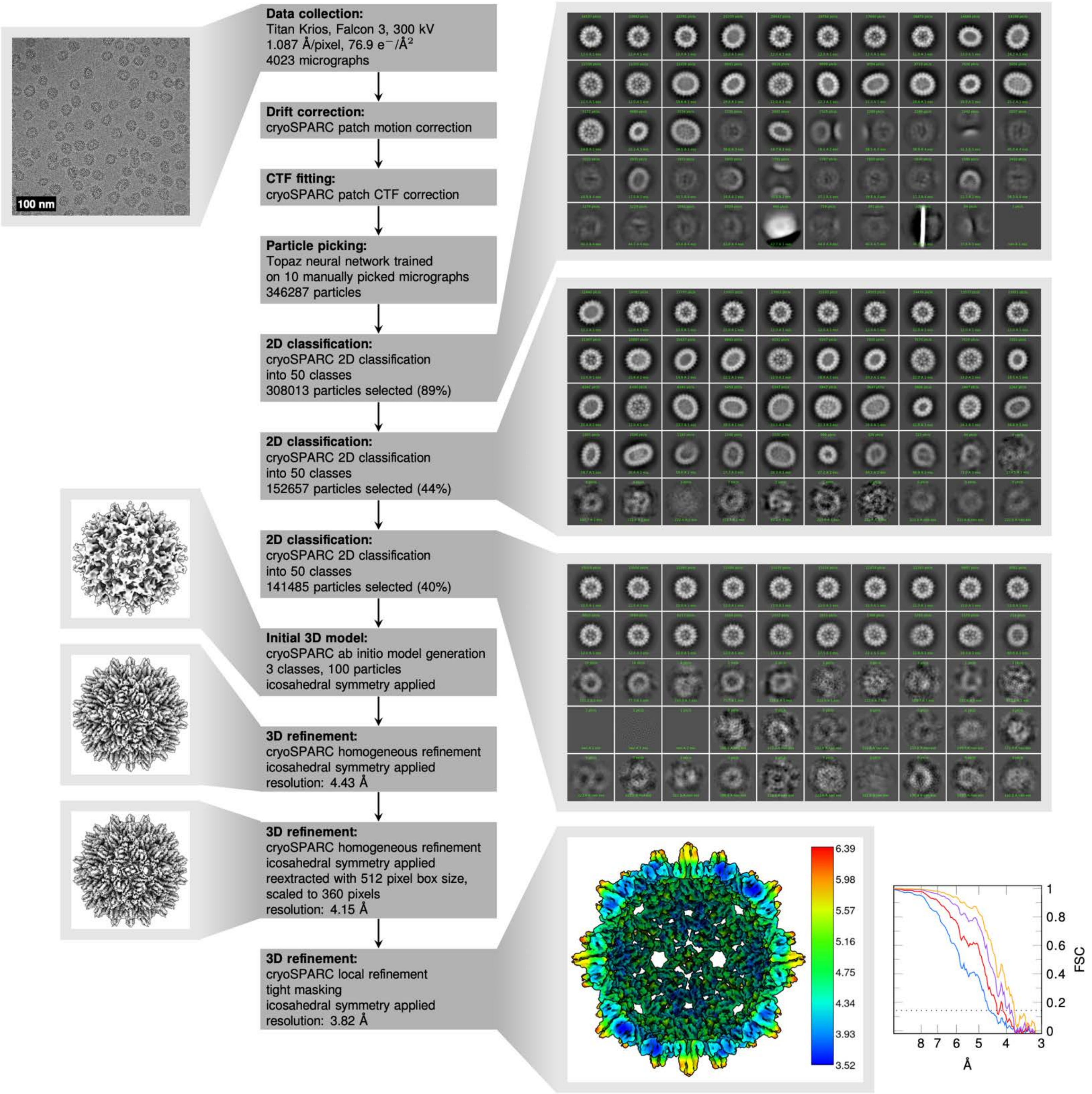

Figure S 13: **Cryo-EM image processing scheme** for the ACNDV capsid pH shift experiment. Processing scheme for ACNDV capsid at pH 5.5. The Fourier shell correlation is plotted for the map without masking (blue), with a spherical mask (red), with a loose mask (purple), and with a tight mask (orange).

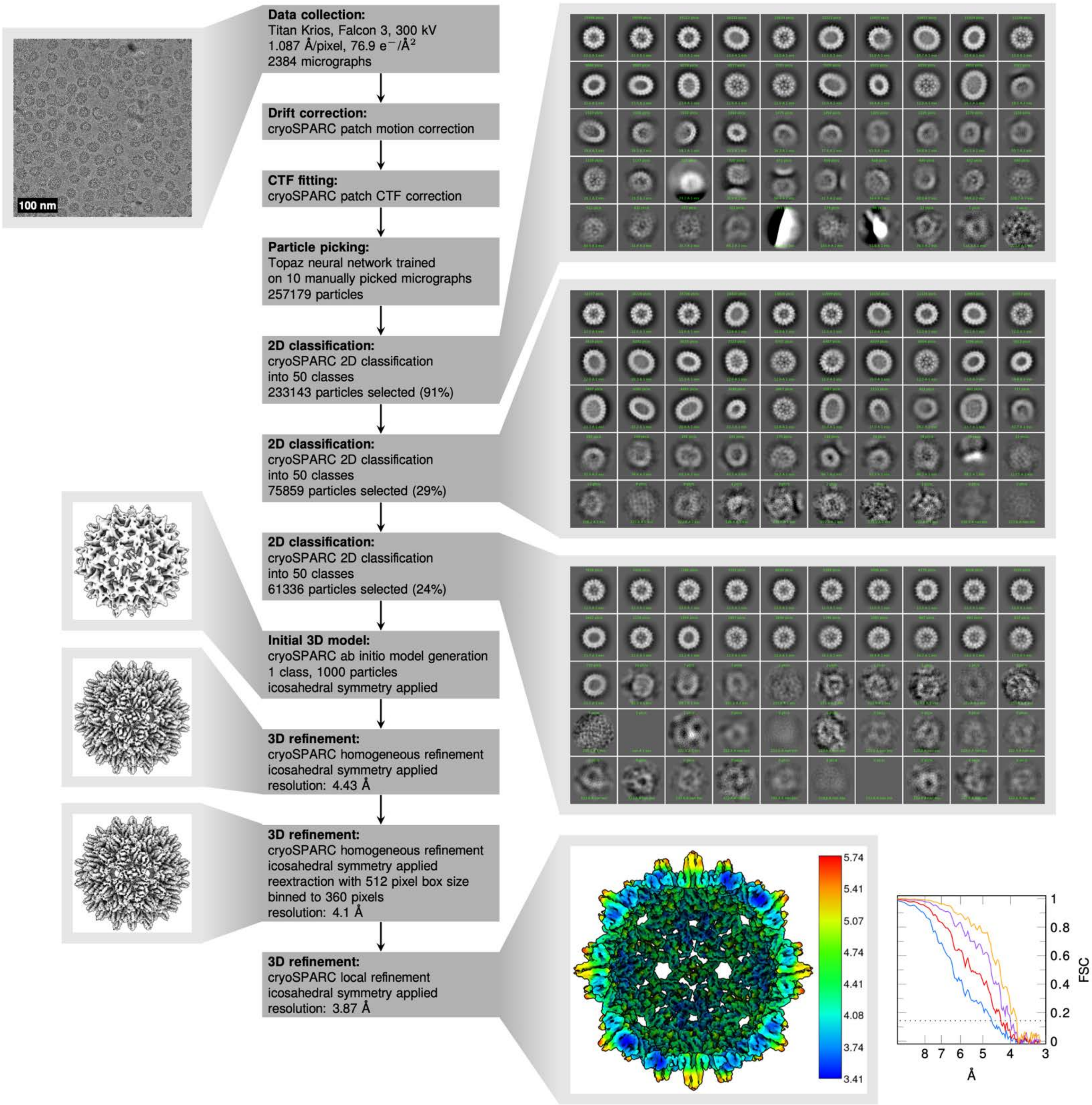

Figure S 14: **Cryo-EM image processing scheme** for the ACNDV capsid pH shift experiment. Processing scheme for ACNDV capsid at pH 7.5 after shift to pH 5.5. The Fourier shell correlation is plotted for the map without masking (blue), with a spherical mask (red), with a loose mask (purple), and with a tight mask (orange).

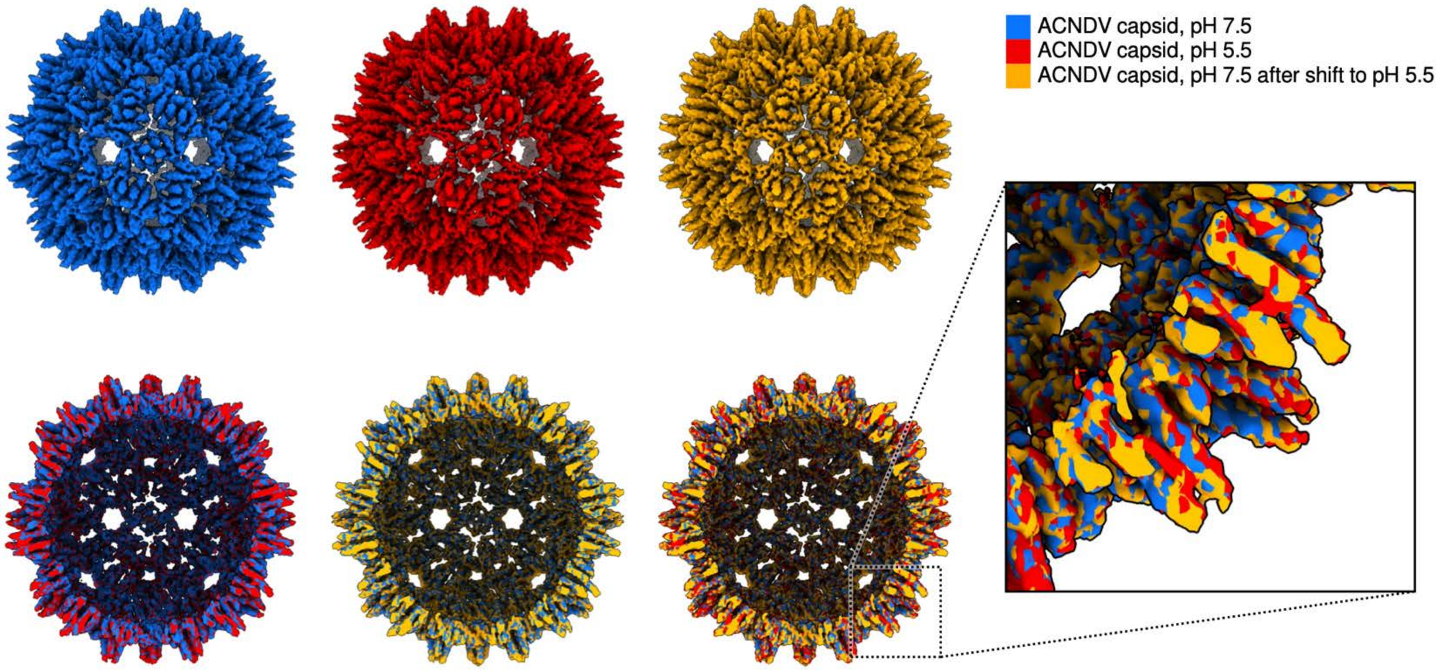

Figure S 15: **The particle diameter does not vary with change of pH.** Cryo-EM maps reconstructed using strictly comparable methodology at pH 7.5 (blue), pH 5.5 (red), and at pH 7.5 after a shift to pH 5.5 (orange) were aligned and show negligible structural differences.

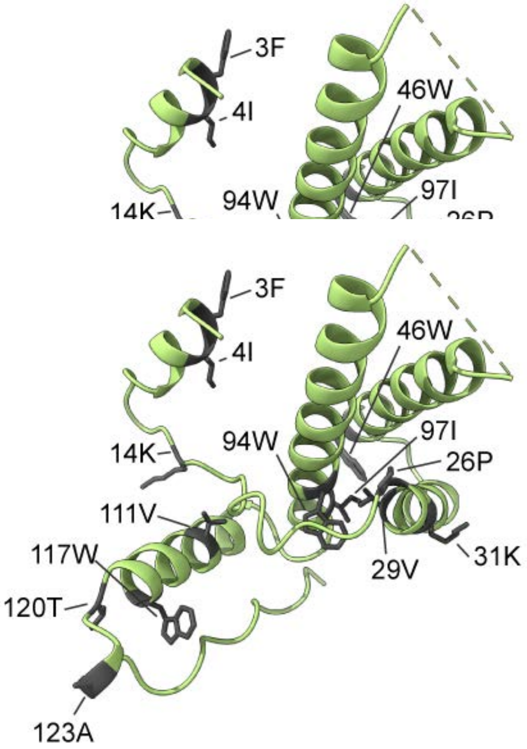

Figure S 16: **NMR peak broadening in ACNDV Cp**. Chain A of ACNDV Cp at pH 7.5 is shown with residues for which peaks are broadened (see Table S 6) displayed in gray.

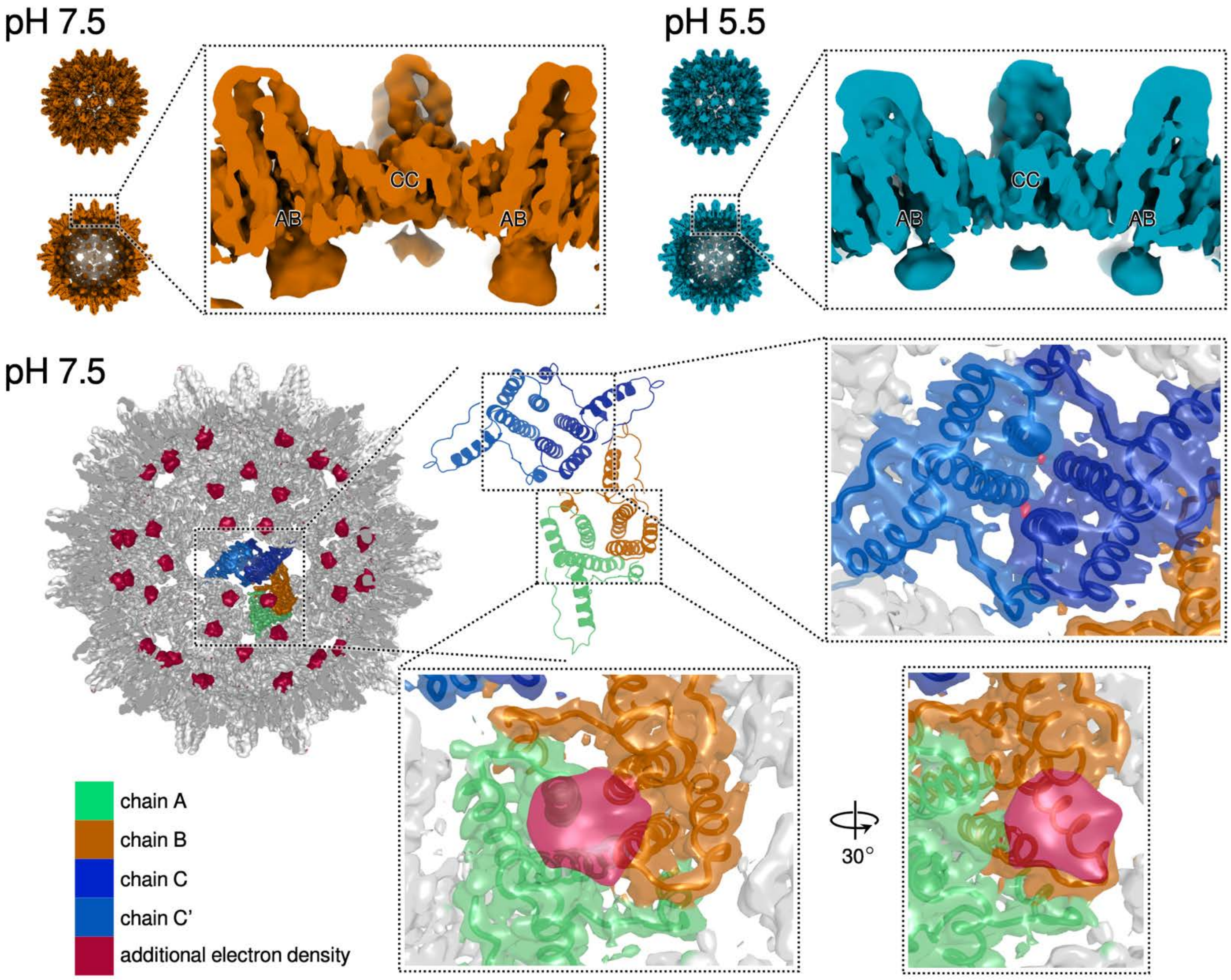

Figure S 17) Local resolution filtered maps of ACNDV**. Additional density arises inside the capsid shell attributed to nucleic acids and the CTD.** Local resolution filtered maps of ACNDV capsid at pH 7.5 (cyan) and pH 5.5 (orange) show additional density below the AB, but not the CC dimer. The connection between the spike helices can be visualized in local resolution filtered maps. Additional electron density is localized directly below the spike helices of the AB dimers, but absent below the CC dimers.

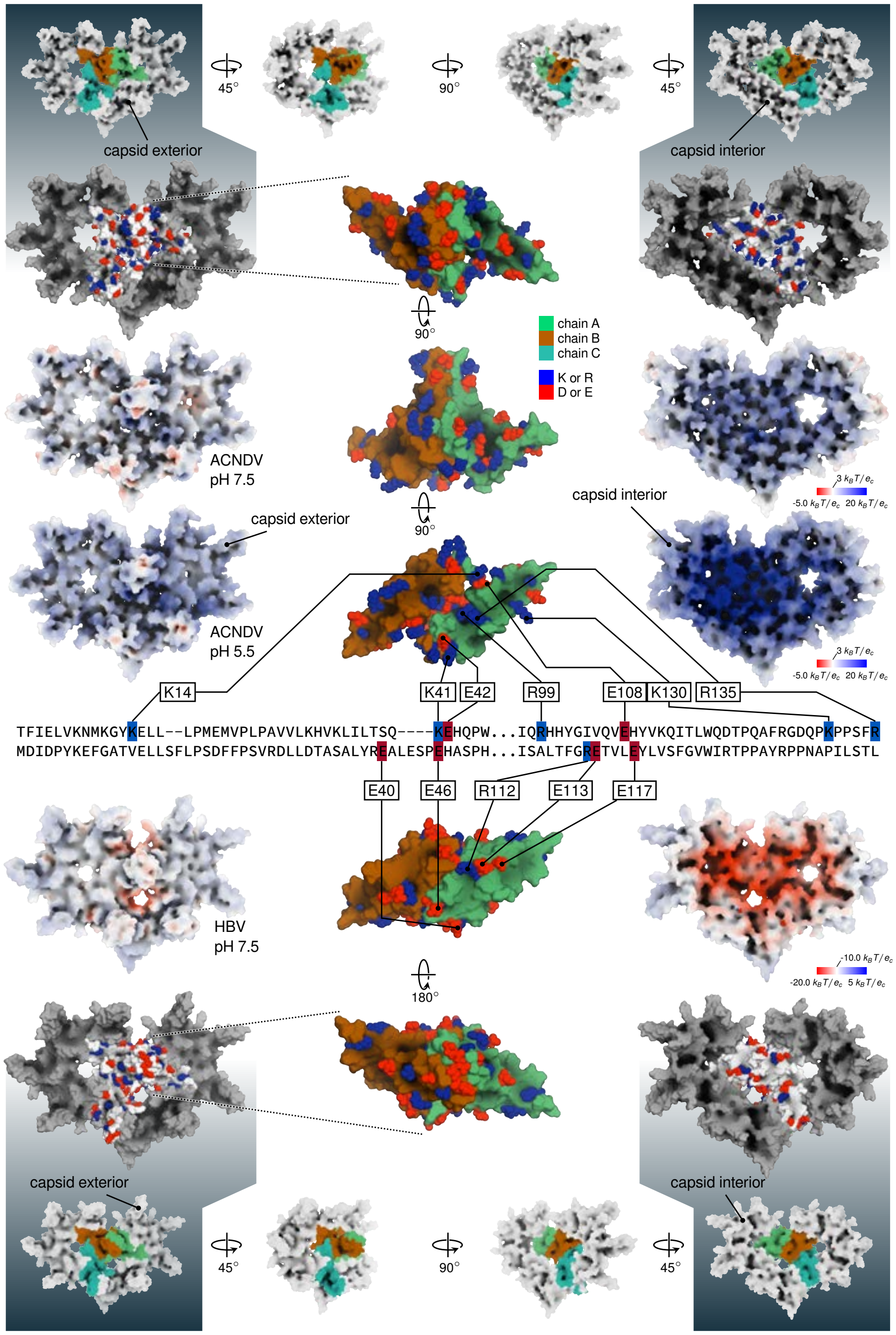

Figure S 18: **The inner surface of the ACNDV capsid carries more positive charge than the inner surface of HBV capsid.** Comparison of the inner and outer surface of ACNDV and HBV capsid (HBV Cp T=3 AB dimer, pdb 6ui6 [13]). The distribution of acidic/negatively charged (E and D, red) and basic/positively charged residues (R and K, blue), and the electrostatic potential maps are shown. Electrostatic potential maps at pH 7.5 and at pH 5.5 are shown for ACNDV. The electrostatic potential map for HBV capsid was calculated at pH 7.5. The PDB2PQR server using the AMBER forcefield in conjunction with PROPKA was used to assign the protonation state at the provided pH. The surface potentials were visualized in PyMOL. A structure-guided alignment of ACNDV and HBV capsid protein is shown to identify structurally equivalent charged residues in the respective capsid proteins.

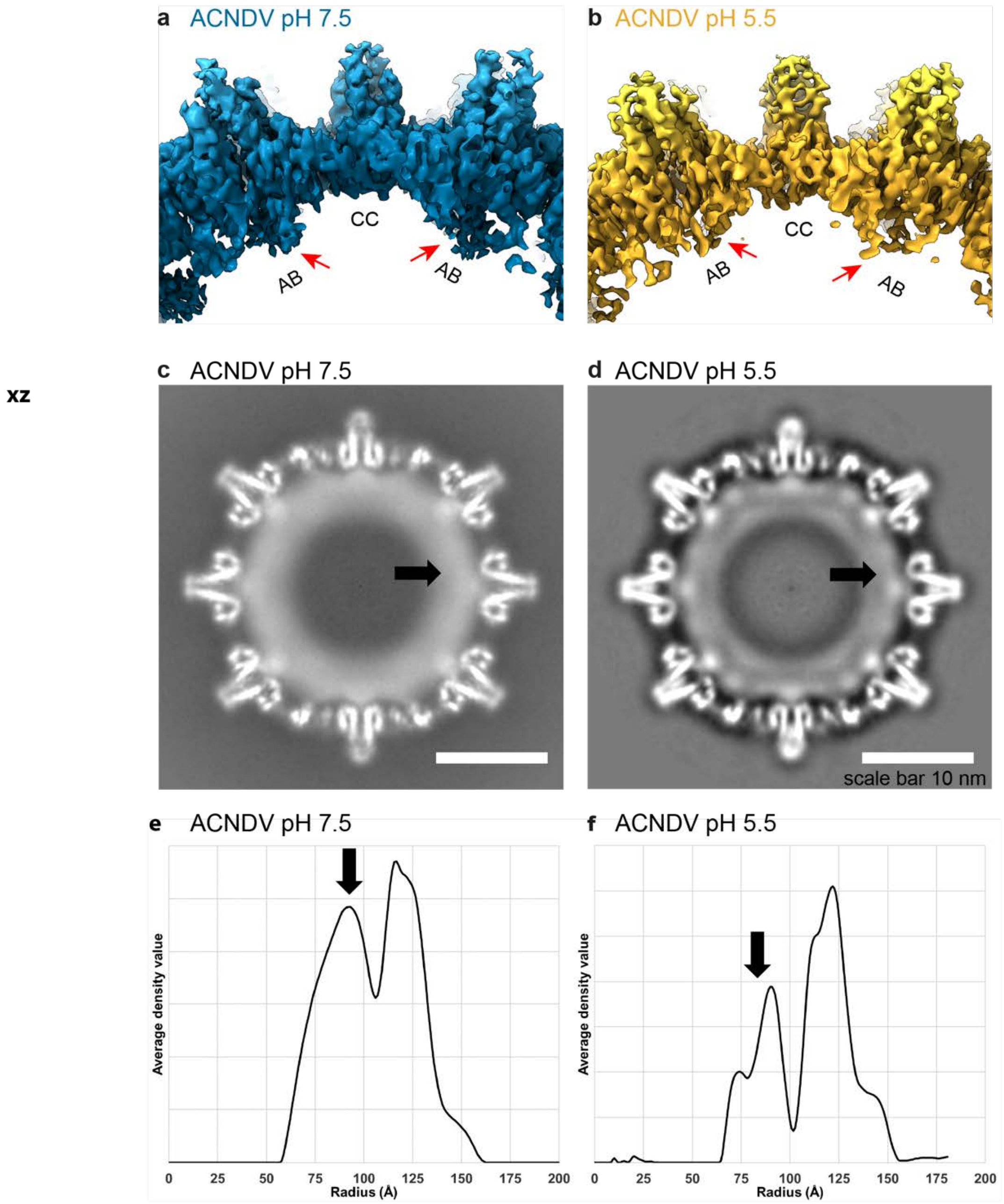

Figure S 19: **Unassigned density below AB dimers and density distribution inside the capsid shell. a)** Cryo-EM density map section of the ACNDV capsid particle at pH 7.5. Non-assigned electron density under the AB dimers (indicated with red arrows) could originate from partially localized RNA that was enclosed into the capsid during recombinant expression in *E.coli* or from the flexible C-terminus of the capsid protein. No additional map density was found under CC dimers **(b)** Unassigned map density below AB dimers of pH 5.5 ACNDV capsids. Small blobs were hidden in both maps with the ChimeraX Hide Dust tool [10]. **c)** Central slice of the density of the ACNDV capsid at pH 7.5. **d)** Central slice of the density of the ACNDV capsid at pH 5.5. **(e)** Radial density plot of ACNDV capsid at pH 7.5 shown in C. **f)** Radial density plot of ACNDV capsid at pH 5.5 shown in D (arrows indicate density shell inside the capsid attributed nucleic acids binding to the CTD).

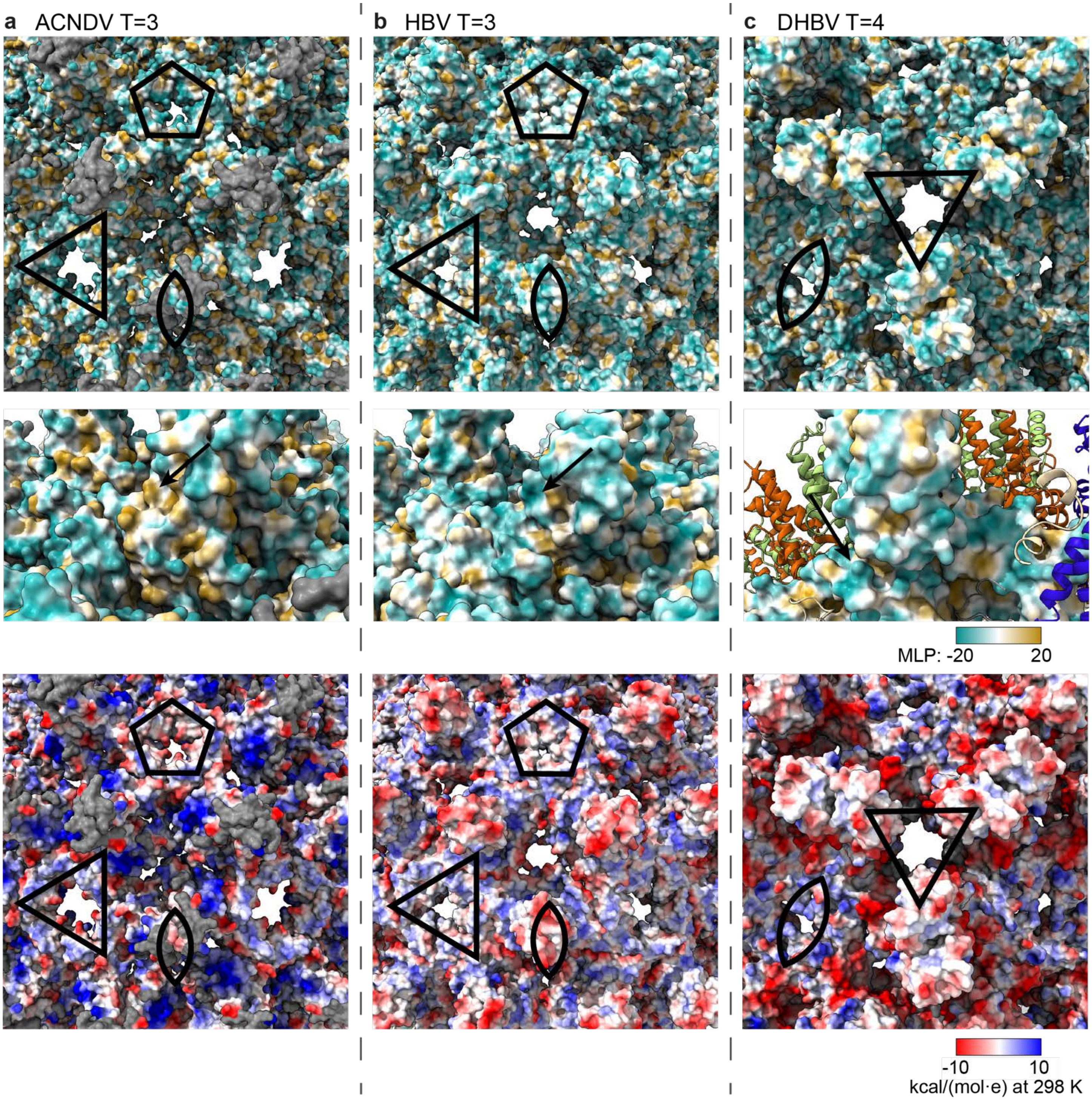

Figure S 20: **Surface properties** of **a)** ACNDV Cp, **b)** HBV Cp (pdb 6ui6) and **)** DHBV Cp (pdb 6ygh). Molecular lipophilicity potentials (mlp command in ChimeraX [14]) are shown in the first two rows with most hydrophilic regions colored in cyan and most lipophilic regions in gold. Symmetry axes are marked with black symbols. Black arrows indicate different MLP values in ACNDV Cp compared to HBV Cp and DHBV Cp. The bottom row shows electrostatic potential values (coulombic in ChimeraX [14]) with negative electrostatic potentials in red and positive ones in blue. The unresolved spikes residues of ACNDV Cp are colored gray.

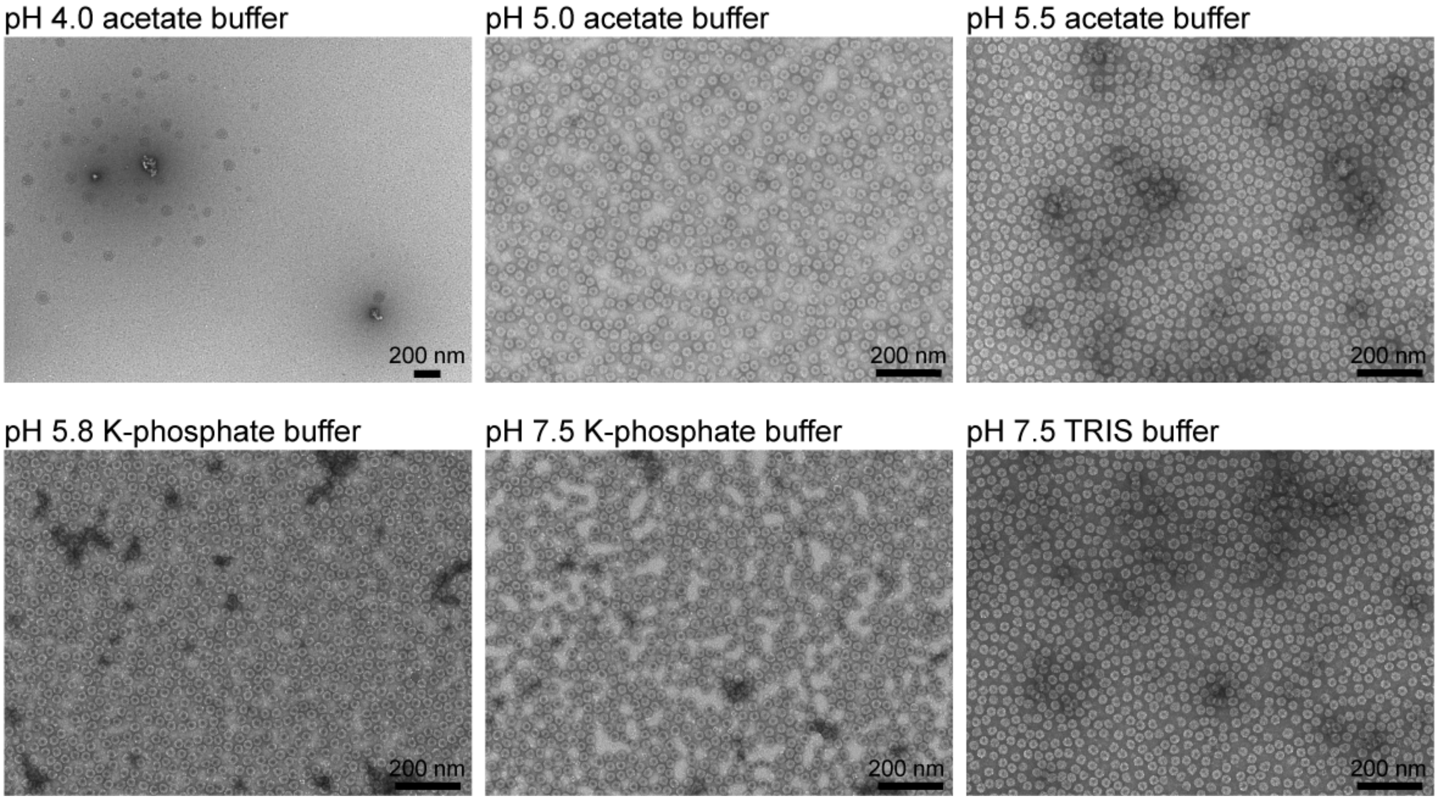

Figure S 21: **ACNDV Cp particles at different pH values.** Sample preparation is described in b. “Low pH screening”.

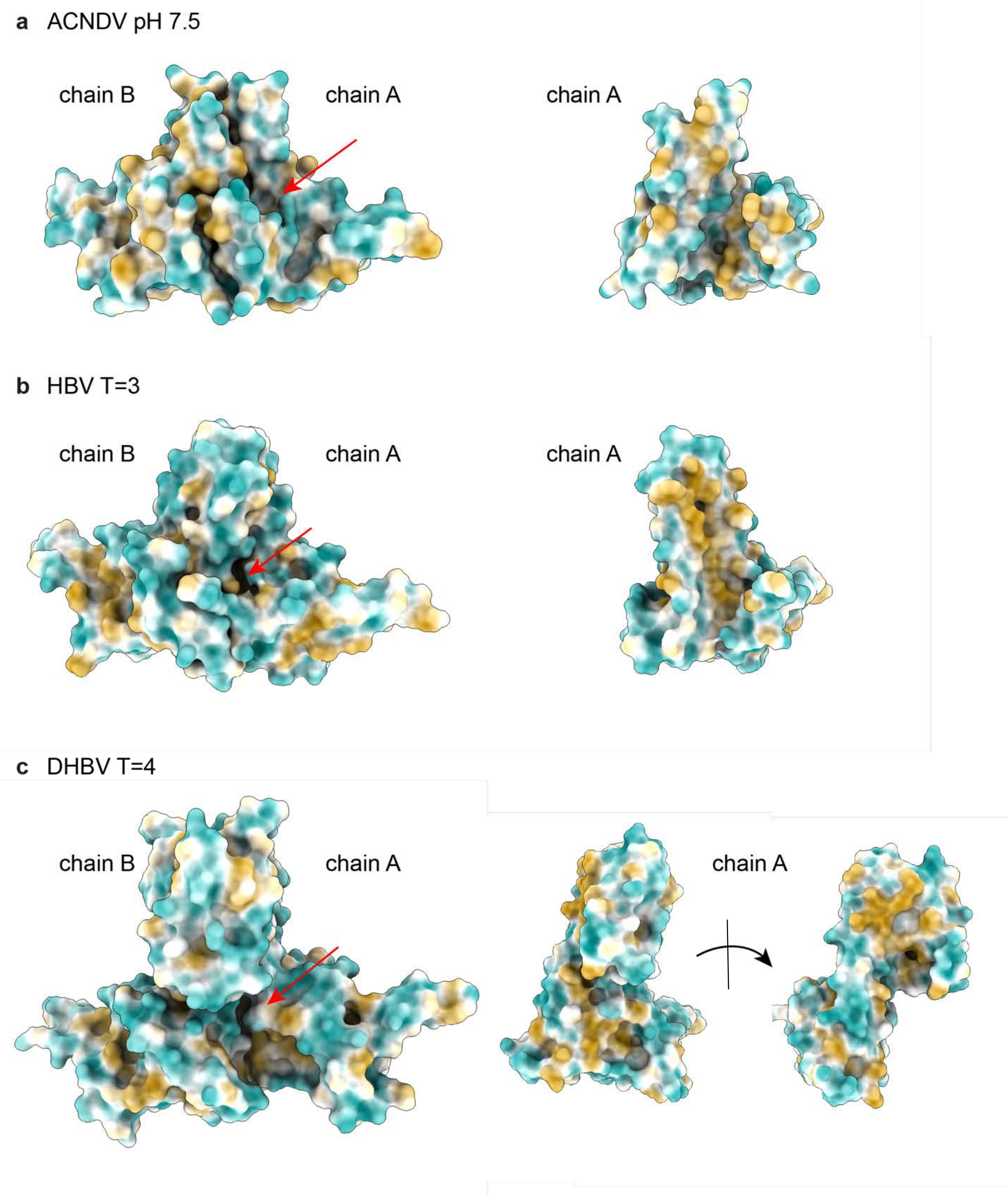

Figure S 22: **Hydrophobic pocket.** Surface representation of **(a)** ACNDV, **(b)** HBV (pdb 6ui6), and **(c)** DHBV (pdb 6ygh) capsid AB dimers and A monomers where cyan marks hydrophilic and gold hydrophobic patches (mlp command in ChimeraX [10]). The hydrophobic pocket of HBV Cp has been characterized (red arrow) [17, 18]. For ACNDV Cp and DHBV Cp, the analogous parts of the protein are also indicated by red arrows but no large hydrophobic area can be identified in between the spike helices.

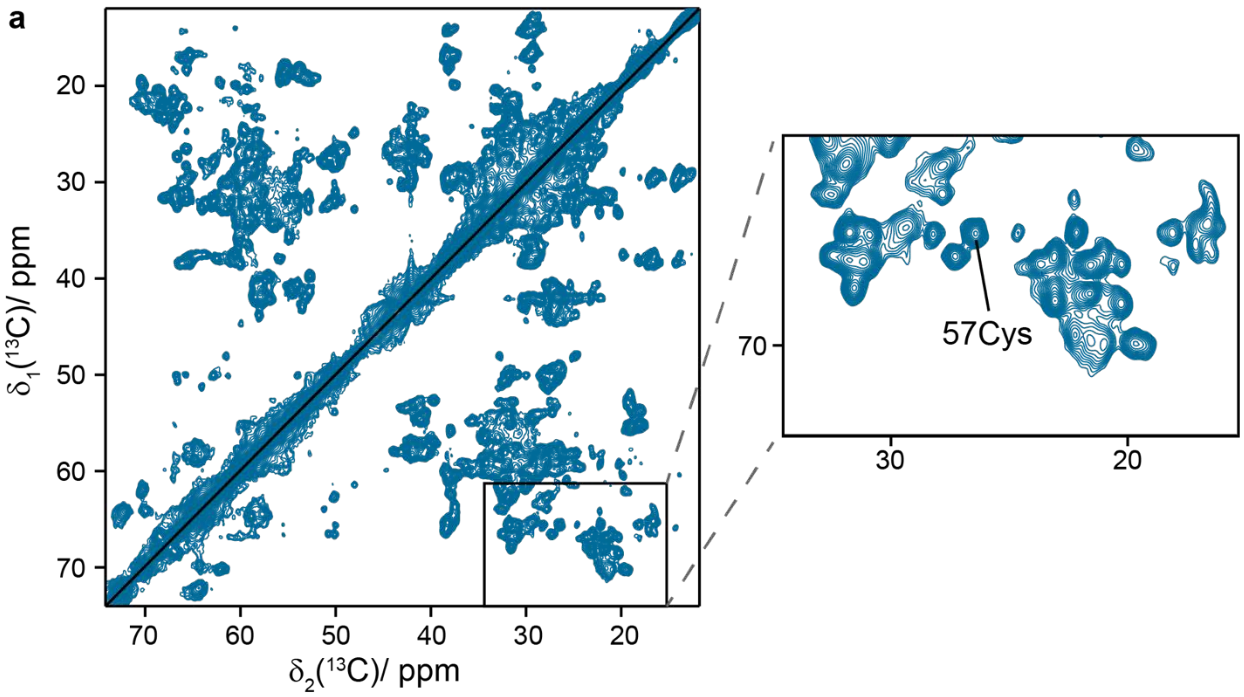

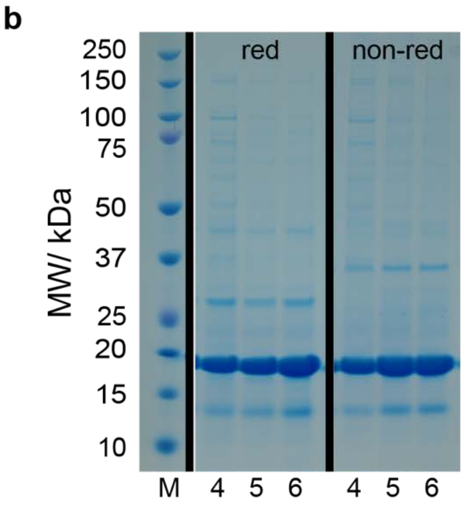

Figure S 23: **Reduced cysteines in ACNDV Cp. a)** DARR spectrum of ACNDV capsids at pH 7.5 and zoom onto peak Cα-Cβ 57Cys. The Cβ chemical shift of 26.5 ppm is characteristic of reduced Cys [19]. The sample preparation of this sample is described in "(a) Uniformly ^{13}C-^{15}N labelled ACNDV capsids at pH 7.5 for NMR measurements" and the 57Cys chemical shift is representative for all measured NMR spectra. **b)** The SDS-PAGE of ACNDV capsids that were produced entirely without DTT (dithiothreitol) as described in "(i) Uniformly ^{13}C-^{15}N labelled ACNDV capsids sample without DTT (dithiothreitol)". Fractions 4, 5, and 6 of the sucrose density gradient were loaded onto the gel once with a buffer that contained DTT (labelled in the image with "red") and once with a non-reducing buffer that contained no DTT (labelled in the image with "non-red"). ACNDV capsid protein appears at 19.8 kDa. If dimers exist, they would only be present in small amounts (band at 39.6 kDa in "non-red" lanes). Here, non-concurrent parts of one gel were joint as indicated by black vertical lines.

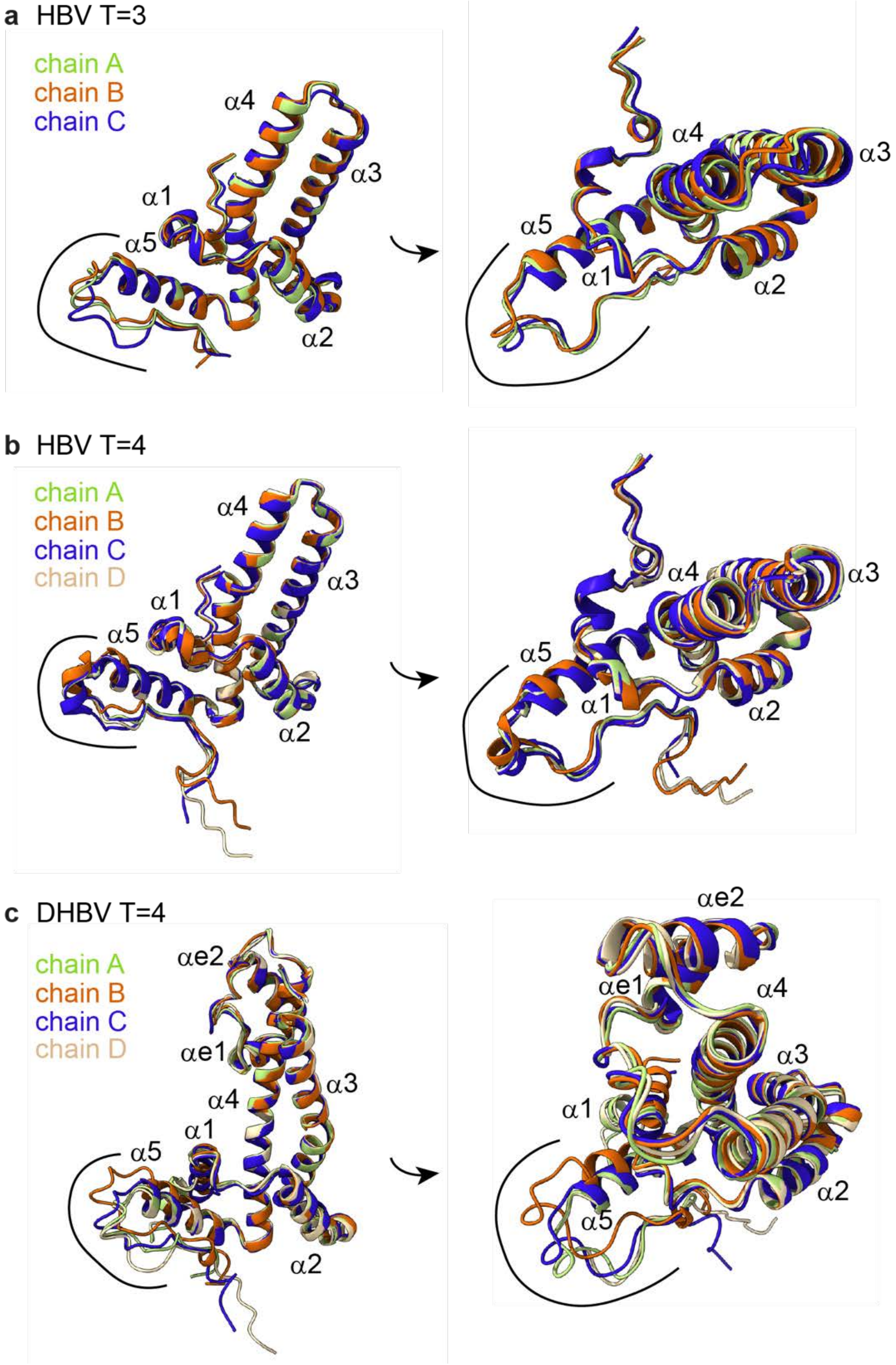

Figure S 24: **Chains of HBV Cp T=3, HBV Cp T=4, and DHBV Cp T=4** were superimposed with the matchmaker command implemented in ChimeraX [10-12]. **a)** Superposition of the three chains A, B, and C of the asymmetric unit of the HBV T=3 capsid (pdb 6ui6) [13]. **b)** Superposition of the four chains A, B, C, and D of the asymmetric unit of the HBV T=4 capsid (pdb 6htx) [15]. **c)** Superposition of the four chains A, B, C, and D of the DHBV capsid (pdb 6ygh) [14]. For HBV T=3, HBV T=4, and DHBV Cp the chains deviate in the hand region angle (indicated with black lines).

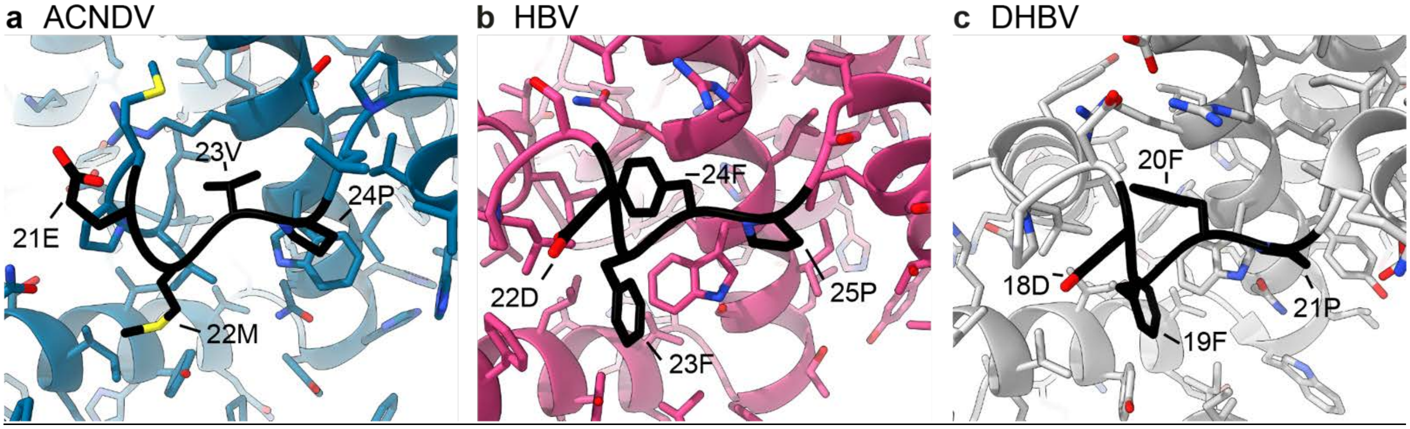

Figure S 25: **a) EMVP sequence in ACNDV Cp, b) DFFP motif in HBV Cp (pdb 6ui6), and c) DFFP motif in DHBV Cp (pdb 6ygh).**

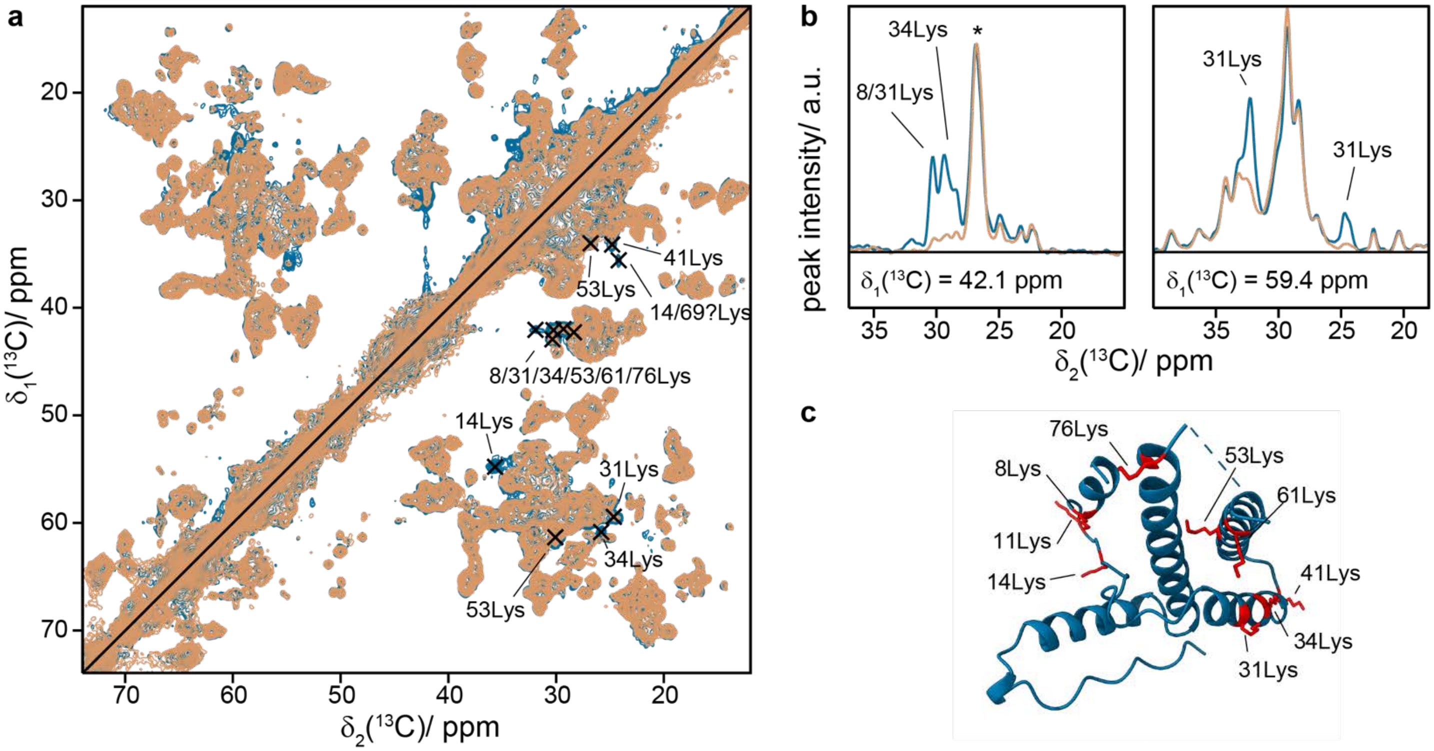

Figure S 26: **Lysine peaks with small intensity. a)** Overlay of two ACNDV capsid protein pH 7.5 DARR spectra. In light brown, a sample is displayed that was obtained with supposedly identical expression protocol as the sample in blue. All assigned lysine residues show significantly decreased cross peak intensities in the pink spectrum. A question mark (?) refers to a tentative assignment of a spike residue (see Figure 4). **b)** Two selected traces show the decreased peak intensities of lysine residues. The traces were scaled to same intensities of the peak labelled with an asterisk (*). **c)** Chain A of the ACNDV capsid protein with lysine residues highlighted in red that display decreased NMR peak intensity. This behavior of decreased lysine residue sensitivity was observed for 3 out of 6 ACNDV capsid protein expressions and the cause of the effect is unknown.

References

1. Liebschner, D., et al., *Macromolecular structure determination using X-rays, neutrons and electrons: recent developments in Phenix.* Acta Crystallographica Section D-Structural Biology, 2019. **75**: p. 861-877.
2. Chen, V.B., et al., *MolProbity: all-atom structure validation for macromolecular crystallography.* Acta Crystallogr D Biol Crystallogr, 2010. **66**(Pt 1): p. 12-21.
3. Prisant, M.G., et al., *New tools in MolProbity validation: CaBLAM for CryoEM backbone, UnDowser to rethink "waters," and NGL Viewer to recapture online 3D graphics.* Protein Sci, 2020. **29**(1): p. 315-329.
4. Williams, C.J., et al., *MolProbity: More and better reference data for improved all-atom structure validation.* Protein Sci, 2018. **27**(1): p. 293-315.
5. Afonine, P.V., et al., *New tools for the analysis and validation of cryo-EM maps and atomic models.* Acta Crystallogr D Struct Biol, 2018. **74**(Pt 9): p. 814-840.
6. Smith-Penzel, S., *Solid-state NMR under fast magic-angle spinning (90-150 kHz) for biological applications*, in *Dep. of Chemistry and Applied Biosciences*. 2019, ETH Zurich: Zurich. p. 295.
7. Ulrich, E.L., et al., *BioMagResBank.* Nucleic Acids Res, 2008. **36**(Database issue): p. D402-8.
8. Wiegand, T., et al., *Asparagine and Glutamine Side-Chains and Ladders in HET-s(218-289) Amyloid Fibrils Studied by Fast Magic-Angle Spinning NMR.* Front Mol Biosci, 2020. **7**: p. 582033.
9. Wu, G., C.J. Freure, and E. Verdurand, *Proton Chemical Shift Tensors and Hydrogen Bond Geometry: A 1H−2H Dipolar NMR Study of the Water Molecule in Crystalline Hydrates.* Journal of the American Chemical Society, 1998. **120**(50): p. 13187-13193.
10. Pettersen, E.F., et al., *UCSF ChimeraX: Structure visualization for researchers, educators, and developers.* Protein Sci, 2021. **30**(1): p. 70-82.
11. Meng, E.C., et al., *Tools for integrated sequence-structure analysis with UCSF Chimera.* BMC Bioinformatics, 2006. **7**: p. 339.
12. Goddard, T.D., et al., *UCSF ChimeraX: Meeting modern challenges in visualization and analysis.* Protein Sci, 2018. **27**(1): p. 14-25.
13. Wu, W., et al., *Expression of quasi-equivalence and capsid dimorphism in the Hepadnaviridae.* PLoS Comput Biol, 2020. **16**(4): p. e1007782.
14. Makbul, C., M. Nassal, and B. Bottcher, *Slowly folding surface extension in the prototypic avian hepatitis B virus capsid governs stability.* Elife, 2020. **9**.
15. Böttcher, B. and M. Nassal, *Structure of Mutant Hepatitis B Core Protein Capsids with Premature Secretion Phenotype.* J Mol Biol, 2018. **430**(24): p. 4941-4954.
16. Dill, J.A., et al., *Distinct Viral Lineages from Fish and Amphibians Reveal the Complex Evolutionary History of Hepadnaviruses.* J Virol, 2016. **90**(17): p. 7920-33.
17. Lecoq, L., et al., *A pocket-factor-triggered conformational switch in the hepatitis B virus capsid.* Proc Natl Acad Sci U S A, 2021. **118**(17).
18. Makbul, C., et al., *Binding of a Pocket Factor to Hepatitis B Virus Capsids Changes the Rotamer Conformation of Phenylalanine 97.* Viruses, 2021. **13**(11).
19. Sharma, D. and K. Rajarathnam, *13C NMR chemical shifts can predict disulfide bond formation.* J Biomol NMR, 2000. **18**(2): p. 165-71.
