# Supplementary material for: Structural conservation of HBV-like capsid proteins over hundreds of millions of years despite the shift from non-enveloped to enveloped life-style
Source: Nat Commun. 2023 Mar 22;14:1574. doi: 10.1038/s41467-023-37068-w (PMC10033635; doi:10.1038/s41467-023-37068-w)
Supplement: Supplementary file 2 — Reporting Summary [file 41467_2023_37068_MOESM2_ESM.pdf]

nature portfolio

Corresponding author(s): Beat H Meier

Last updated by author(s): 2022/11/25

Reporting Summary

Nature Portfolio wishes to improve the reproducibility of the work that we publish. This form provides structure for consistency and transparency in reporting. For further information on Nature Portfolio policies, see our Editorial Policies and the Editorial Policy Checklist.

Statistics

For all statistical analyses, confirm that the following items are present in the figure legend, table legend, main text, or Methods section.

n/a	Confirmed	
☐	☒	The exact sample size (*n*) for each experimental group/condition, given as a discrete number and unit of measurement
☒	☐	A statement on whether measurements were taken from distinct samples or whether the same sample was measured repeatedly
☒	☐	The statistical test(s) used AND whether they are one- or two-sided<br>*Only common tests should be described solely by name; describe more complex techniques in the Methods section.*
☒	☐	A description of all covariates tested
☒	☐	A description of any assumptions or corrections, such as tests of normality and adjustment for multiple comparisons
☒	☐	A full description of the statistical parameters including central tendency (e.g. means) or other basic estimates (e.g. regression coefficient) AND variation (e.g. standard deviation) or associated estimates of uncertainty (e.g. confidence intervals)
☒	☐	For null hypothesis testing, the test statistic (e.g. *F*, *t*, *r*) with confidence intervals, effect sizes, degrees of freedom and *P* value noted<br>*Give P values as exact values whenever suitable.*
☒	☐	For Bayesian analysis, information on the choice of priors and Markov chain Monte Carlo settings
☒	☐	For hierarchical and complex designs, identification of the appropriate level for tests and full reporting of outcomes
☒	☐	Estimates of effect sizes (e.g. Cohen's *d*, Pearson's *r*), indicating how they were calculated

Our web collection on statistics for biologists contains articles on many of the points above.

Software and code

Policy information about availability of computer code

Data collection	NMR: Topspin 3.5 (Bruker corporation) cryo-EM: EPU1.0/1.10 data acquisition software
Data analysis	NMR: CcpNmr V2, cryo-EM: Coot 0.8.9, UCSF ChimeraX 1.4, Phenix 1.20.1-4487, ISOLDE 1.0b4.dev0, PyMOL 2.5.2, hole2 (V2) crYOLO(1.7.6), Gctf(v1.06),MotionCorr2(v1.06), Relion(3.1), Phenix(1.20.1-4487), CryoSPARC(v3.3.1), Topaz(0.2.4), ChimeraX(1.4), Pymol(2.5.2), ISOLDE (1.0b4.dev0)

For manuscripts utilizing custom algorithms or software that are central to the research but not yet described in published literature, software must be made available to editors and reviewers. We strongly encourage code deposition in a community repository (e.g. GitHub). See the Nature Portfolio guidelines for submitting code & software for further information.

Data

Policy information about availability of data

All manuscripts must include a data availability statement. This statement should provide the following information, where applicable:

- Accession codes, unique identifiers, or web links for publicly available datasets
- A description of any restrictions on data availability
- For clinical datasets or third party data, please ensure that the statement adheres to our policy

Chemical shifts of 13C, 15N, and 1H atoms of ACNDV Cp at pH 7.5 were deposited in the Biological Magnetic Resonance Data Bank under accession number 51506. Cryo-EM maps of ACNDV capsids at pH 7.5 and at pH 5.5 were deposited in the Electron Microscopy Data Bank under accession numbers EMD-15295 (pH 7.5) and EMD- 16371 (pH 5.5) and molecular capsid structures were submitted to the Protein Data Bank under accession numbers 8AAC (pH 7.5) and 8C0O, (pH 5.5). Two-

and 3-dimensional NMR spectra are available upon request.

Human research participants

Policy information about studies involving human research participants and Sex and Gender in Research.

Reporting on sex and gender	N/A
Population characteristics	N/A
Recruitment	N/A
Ethics oversight	N/A

Note that full information on the approval of the study protocol must also be provided in the manuscript.

Field-specific reporting

Please select the one below that is the best fit for your research. If you are not sure, read the appropriate sections before making your selection.

[x] Life sciences   [ ] Behavioural & social sciences   [ ] Ecological, evolutionary & environmental sciences

For a reference copy of the document with all sections, see nature.com/documents/nr-reporting-summary-flat.pdf

Life sciences study design

All studies must disclose on these points even when the disclosure is negative.

Sample size	We have one protein sample, however NMR measures a large ensemble of protein molecules that add up to the NMR signal
Data exclusions	No data were excluded
Replication	Bevor each measurement session, spectrometer parameters were verified by standard tune-up sequences
Randomization	N/A
Blinding	N/A

Reporting for specific materials, systems and methods

We require information from authors about some types of materials, experimental systems and methods used in many studies. Here, indicate whether each material, system or method listed is relevant to your study. If you are not sure if a list item applies to your research, read the appropriate section before selecting a response.

Materials & experimental systems

n/a	Involved in the study	
[x]	[ ]	Antibodies
[x]	[ ]	Eukaryotic cell lines
[x]	[ ]	Palaeontology and archaeology
[x]	[ ]	Animals and other organisms
[x]	[ ]	Clinical data
[x]	[ ]	Dual use research of concern

Methods

n/a	Involved in the study	
[x]	[ ]	ChIP-seq
[x]	[ ]	Flow cytometry
[x]	[ ]	MRI-based neuroimaging
