# Supplementary figures and images for: Structural conservation of HBV-like capsid proteins over hundreds of millions of years despite the shift from non-enveloped to enveloped life-style
Source: Nat Commun. 2023 Mar 22;14:1574. doi: 10.1038/s41467-023-37068-w (PMC10033635; doi:10.1038/s41467-023-37068-w)

Source data:

The uncropped gel of Figure S 23

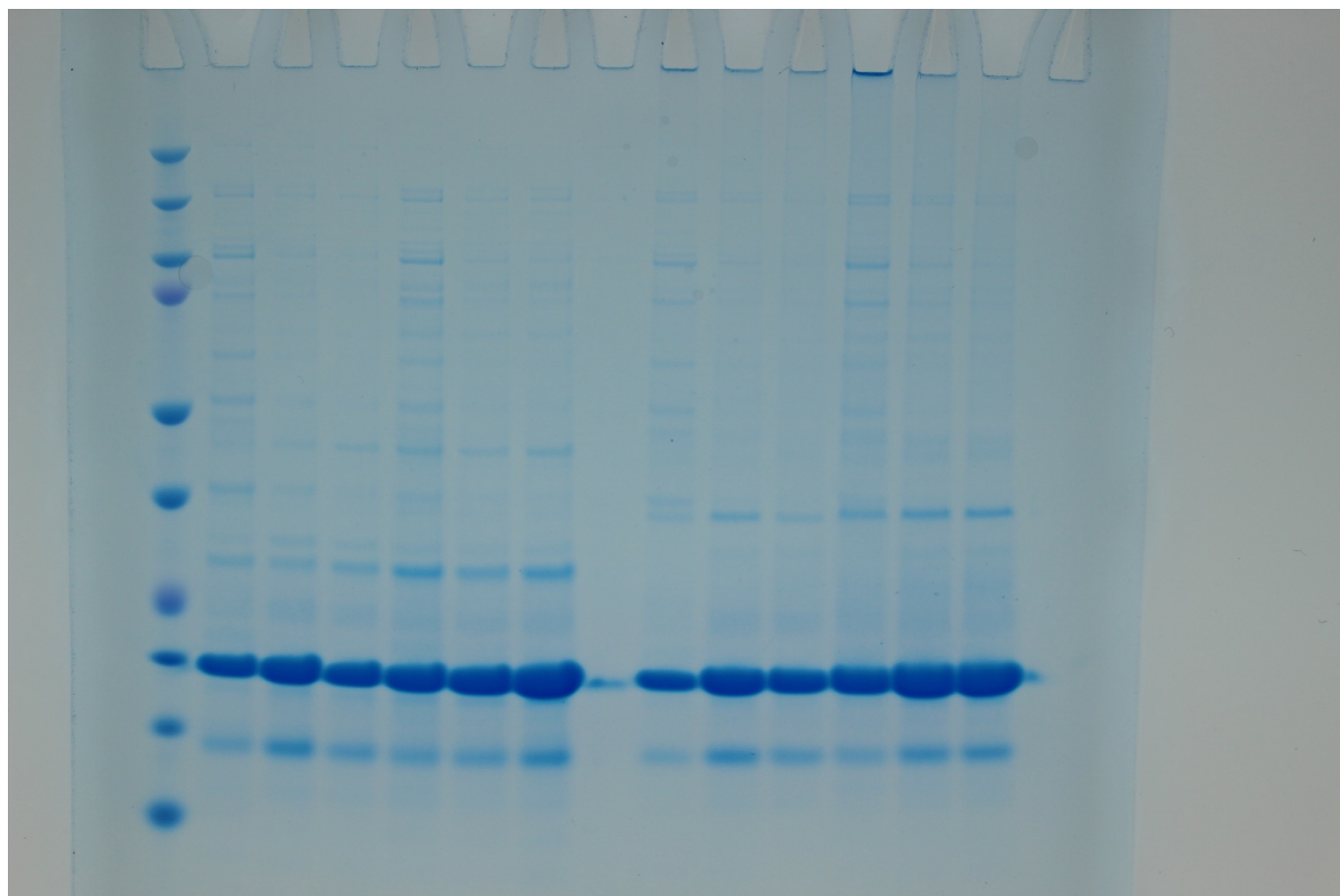

Supplement: Supplementary file 4 — Source Data [file 41467_2023_37068_MOESM4_ESM.zip › Source_data_gel.pdf]
